# Epigenetic Modifications Unlock the Milk Protein Gene Loci during Mouse Mammary Gland Development and Differentiation

**DOI:** 10.1371/journal.pone.0053270

**Published:** 2013-01-02

**Authors:** Monique Rijnkels, Courtneay Freeman-Zadrowski, Joseph Hernandez, Vani Potluri, Liguo Wang, Wei Li, Danielle G. Lemay

**Affiliations:** 1 United States Department of Agriculture/Agriculture Research Service Children’s Nutrition Research Center, Department of Pediatrics-Nutrition, Baylor College of Medicine, Houston, Texas, United States of America; 2 Division of Biostatistics, Dan L Duncan Cancer Center, Department of Molecular and Cellular Biology, Baylor College of Medicine, One Baylor Plaza, Houston, Texas, United States of America; 3 Genome Center, University of California Davis, Davis, California, United States of America; Massachusetts General Hospital, United States of America

## Abstract

**Background:**

Unlike other tissues, development and differentiation of the mammary gland occur mostly after birth. The roles of systemic hormones and local growth factors important for this development and functional differentiation are well-studied. In other tissues, it has been shown that chromatin organization plays a key role in transcriptional regulation and underlies epigenetic regulation during development and differentiation. However, the role of chromatin organization in mammary gland development and differentiation is less well-defined. Here, we have studied the changes in chromatin organization at the milk protein gene loci (casein, whey acidic protein, and others) in the mouse mammary gland before and after functional differentiation.

**Methodology/Principal Findings:**

Distal regulatory elements within the casein gene cluster and whey acidic protein gene region have an open chromatin organization after pubertal development, while proximal promoters only gain open-chromatin marks during pregnancy in conjunction with the major induction of their expression. In contrast, other milk protein genes, such as alpha-lactalbumin, already have an open chromatin organization in the mature virgin gland. Changes in chromatin organization in the casein gene cluster region that are present after puberty persisted after lactation has ceased, while the changes which occurred during pregnancy at the gene promoters were not maintained. In general, mammary gland expressed genes and their regulatory elements exhibit developmental stage- and tissue-specific chromatin organization.

**Conclusions/Significance:**

A progressive gain of epigenetic marks indicative of open/active chromatin on genes marking functional differentiation accompanies the development of the mammary gland. These results support a model in which a chromatin organization is established during pubertal development that is then poised to respond to the systemic hormonal signals of pregnancy and lactation to achieve the full functional capacity of the mammary gland.

## Introduction

Mammary glands are unique to mammals and are the basis of the survival and existence of these species. Unlike most organs and tissues, which undergo most of their development *in utero*, the mammary gland develops primarily after birth. Profound morphological and functional changes take place in the mammary gland during key reproductive stages, and the gland undergoes repeated cycles of functional differentiation with reproduction during the life of a mammal. In the mouse, as in most other mammals, the first postnatal-phase of development occurs at puberty under the influence of puberty-associated hormones and local growth factors [1]–[4]. At the end of this phase the gland is ready for full functional differentiation, which is achieved during the second major developmental phase–pregnancy–ultimately resulting in the production of milk during lactation [2], [5], [6]. Once lactation ceases the gland undergoes remodeling, during which the majority of alveolar epithelial cells that previously secreted milk are lost. These postnatal developmental changes make the mammary gland an ideal model to study the regulation of developmental processes.

Mammary epithelial cell differentiation during puberty and pregnancy is mediated through a succession of cell fate decisions that include changes in expression patterns, increases of cell type-specific expression, and silencing of genes not required for cell identity or cell proliferation [7]–[11]. Corresponding changes have been observed during cell loss and tissue remodeling upon involution [7]–[10]. Over the past decades considerable information has been obtained about the systemic hormones and local growth factors that are important for establishing these expression patterns, as well as the transcription factors involved in their regulation [2], [3], [12]–[18]. However, these factors involved in mammary gland development and tissue-specific gene expression are ubiquitously expressed, and their expression patterns do not explain the tissue- and stage-specificity of their responses (e.g. STAT family of transcription factors [19]; C/EBP [20]; [18], [21]). This suggests that there is yet another level of regulation that contributes to the spatial and temporal regulation needed to produce a fully functional mammary gland.

Chromatin organization plays a key role in transcriptional regulation during development and differentiation [22], [23]. Markers for chromatin organization include the following: 1) different post-translational modifications of the core histones and their N-terminal tails, associated with active or poised/open chromatin or inactive/closed chromatin [24], 2) DNase I hypersensitivity, indicating the presence of open accessible chromatin often marking active regulatory elements [25], and 3) DNA methylation–a key mediator of transcriptional silencing and an overall mark of a closed chromatin organization [26], [27].

Most evolutionary conserved regions (ECRs) outside of coding regions correspond to Distal Regulatory Elements (DREs) [[28]]. DREs serve as anchor points for the binding of proteins that organize DNA/chromatin conformation and architecture, which results in repression or activation of a genomic domain and gene expression [29]–[32]. The presence of open chromatin at evolutionary conserved regions (ECR) corresponds with such a function [33], [34]. Using comparative genome analysis we have previously identified ECRs in the casein gene cluster [35], [36] and a number of distal regulatory elements have been identified for the mouse whey acidic protein gene (Wap) [17], [37]–[39]. In this paper, we investigate developmental stage- and tissue-specific chromatin changes at ECRs near milk protein genes.

Little is known about the chromatin changes that take place during mammary gland development and functional differentiation [40]. Tissue-specific methylation patterns have been reported for the rat beta-casein gene and bovine alpha-s1-casein transgene [41], [42]. Millot and colleagues [17] have described hormone-dependent and tissue-specific DNaseI hypersensitive sites (DHS) in the Wap upstream region, which are conserved between mouse, rat and rabbit [17], [37]. A tissue specific DHS site in the rat Wap promoter identified by Li and Rosen (rHSS1; [38]) was shown to be essential for tissue-specific expression of a rat Wap transgene in mice and the presence of this DHS in the lactating mammary gland of transgenic mice is dependent on the presence of glucocorticoids [38], [39]. Furthermore, binding of STAT5 and NF1 to the rat HSS1 was shown to be required for maximal tissue-specific expression of the rat Wap transgene [16]. Tissue- and developmentally-regulated DHS also have been identified for the ovine beta-lactoglobulin gene promoter [43]. Furthermore, lactogenic hormone induction of casein gene expression in cell culture is linked with changes in histone modifications and recruitment of signal-transducing factors [44]–[47]. Maruyama et al recently reported differences in chromatin organization of a progenitor-enriched and a more luminal human breast epithelial cell population [48]. Together these data indicate a role for mammary tissue-specific and cell-specific chromatin organization. However, temporally-specific chromatin changes during key developmental stages of the mammary gland have not been explored.

Since milk protein genes such as the casein genes and Wap encode the major milk proteins, which are secreted by the functionally differentiated epithelial cells in high amounts during lactation, they serve as markers for functionally differentiated cells. It has been suggested that the coordinated regulation of the casein genes is due to distal regulatory elements located within the casein gene cluster [35], [36] and as mentioned above a number of distal regulatory elements have been identified for the Wap gene. To investigate if chromatin organizational changes are part of the paradigm for the tissue-and developmental-stage specific regulation of gene expression during mammary gland development, we have studied the changes in chromatin organization–DNA methylation, DNaseI hypersensitivity and histone modifications–at genomic regions harboring milk protein genes, including the casein gene cluster and Wap region, during mammary gland development.

## Results

### Chromatin Organization in the Lactating Mammary Gland as Compared to Liver

We hypothesized that chromatin organization is part of the regulation of gene expression in the functionally differentiating mammary gland. Open chromatin structure is expected on regulatory elements of genes expressed during lactation in the lactating mammary gland, while a closed chromatin structure is expected on regulatory elements of non-expressed genes. Additionally, lactation-specific genes are expected to be associated with closed chromatin in non-expressing and non-mammary tissues. Accordingly, we investigated several markers of chromatin structure in milk protein gene genomic regions in lactating or late-pregnant mammary gland tissue and compared this to non-mammary tissue (liver tissue).

#### Histone modifications in the lactating mammary gland as compared to liver

**Figure 1:**
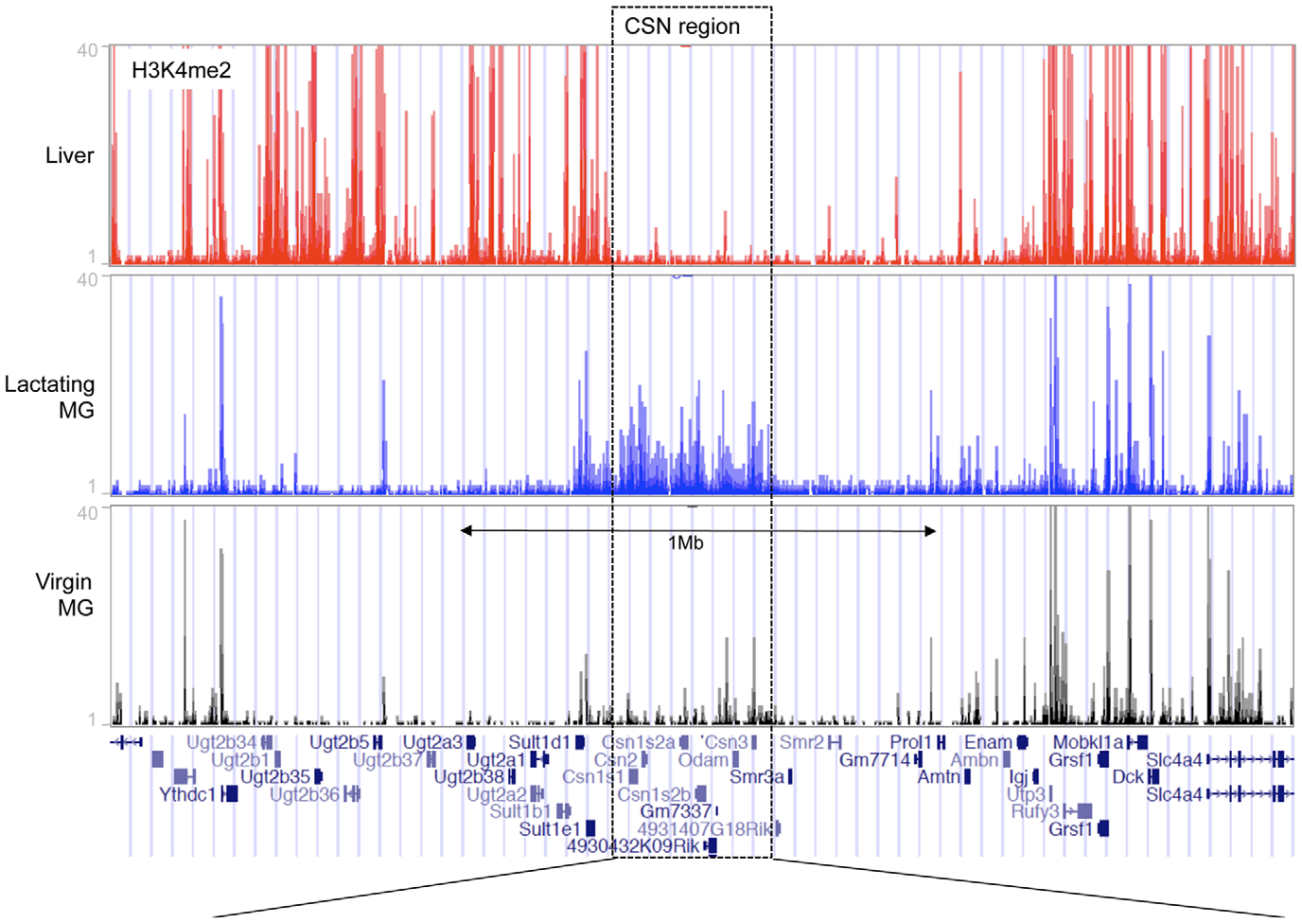
Summary of ChIP-seq for H3K4me2 at the casein gene cluster region and flanking regions. ChIP-seq reads aligned to mouse gene assembly mm9 in the UCSC Genome Browser for H3K4me2 enrichment in liver tissue (red), lactating mammary gland (blue) and mammary epithelial cells (MEC) isolated from 12 week old virgin animals in diestrus (black). The bottom panel displays the locations of annotated genes.

**Figure 2:**
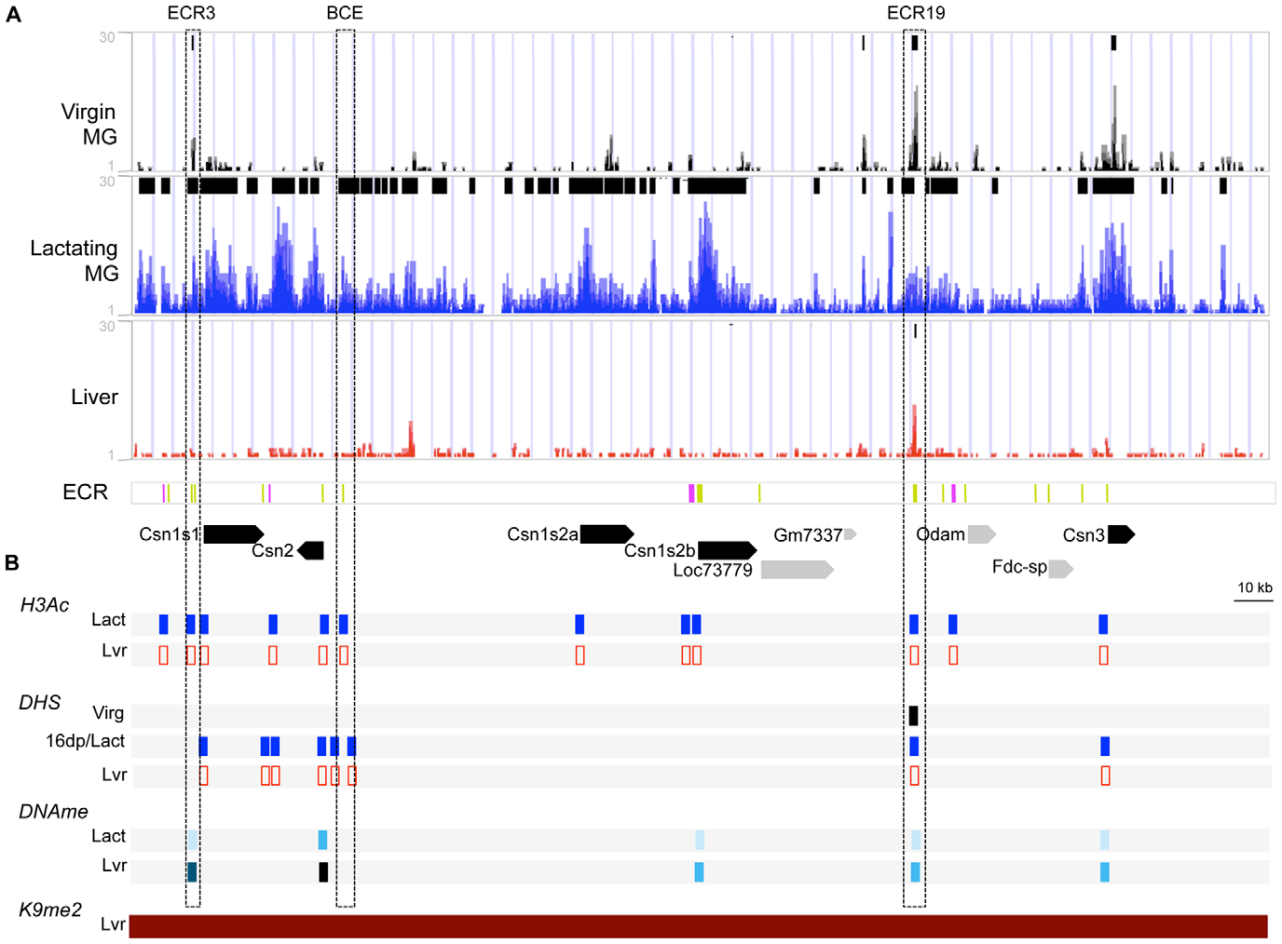
Chromatin markers in the casein gene cluster region in different tissues and developmental stages. This illustration is a close-up of the CSN region from Figure 1. (**A**) ChIP-seq reads for H3K4me2 in MEC isolated from virgin mammary glands at 12 weeks of age, staged at diestrus (**Virgin MG**, black), in lactating mammary glands (**Lactating MG**, blue) and in liver tissue (**Liver**, red). A summary of peaks identified by MAC and SICER are at the top of each of the Virgin MG, Lactating MG, and Liver panels. In the evolutionary conserved region (**ECR**) panel, ECRs are shown in green; ECR1, 6 16 and 21 are indicated in pink. In the bottom panel, the locations of the casein genes (black) and non-casein genes (gray) are indicated by solid arrows denoting the direction of transcription. (**B**) Summary of markers of epigenetic regulation in the CSN region. (***H3Ac***) Summary of results of ChIP for H3-acetylation on lactating mammary gland (**Lact**, blue) and liver tissue (**Lvr**, red). Sites investigated are indicated by rectangles: filled rectangles, H3AC enriched; open rectangle, non-enriched (See figure S3). (***DHS***) Summary of DNaseI Hypersensitivity analysis in MEC isolated from from virgin mammary glands at 12 weeks of age, staged at diestrus (**Virg**, black), in lactating mammary gland (**Lact**, blue) and liver tissue (**Lvr**, red). Sites investigated are indicated by rectangles: filled rectangle, DHS; open rectangle, non-DHS (see Fig. S4). (***DNAme***) Summary of results of DNA methylation analysis on lactating mammary gland (**Lact**) and liver tissue (**Lvr**) (see also Fig. 5). Levels of DNA methylation are based on Fig. 5A and Table S2 and are color coded as follows: 0–20% lightest blue, 21–40% light blue, 41–60% blue, 61–80% dark blue and 80–100% black; open rectangles: not analyzed. (***K9me2***) Summary of ChIP-chip for H3K9me2, a histone modification associated with closed chromatin, on liver tissue from [62]; red filled rectangle indicates enrichment of H3K9me2 in liver tissue.

**Figure 3:**
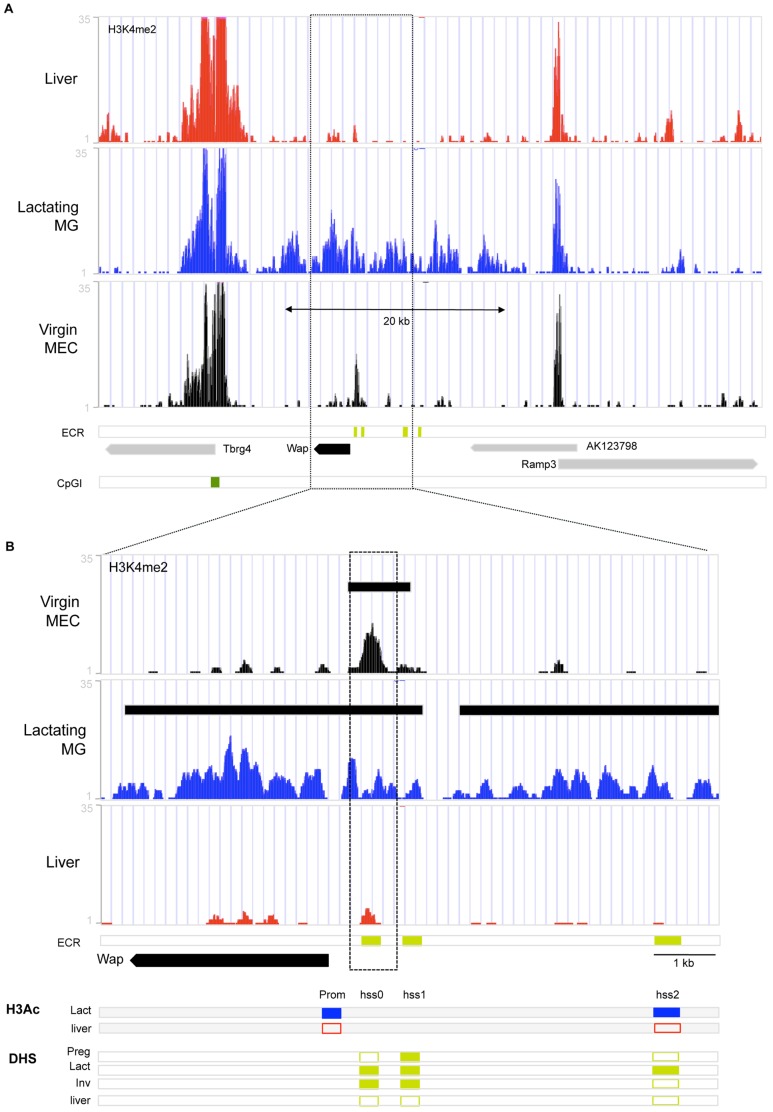
Chromatin organization at genomic region around Whey Acidic Protein gene (Wap). Summary of markers of chromatin organization aligned to the Wap region in mouse genome assembly mm9 in the UCSC Genome Browser. (**A–B**) ChIP-seq reads for H3K4me2 in liver tissue (**Liver**, red), lactating mammary gland (**Lactating MG**, blue) and mammary epithelial cells isolated from 12 week virgin mammary glands (**Virgin MEC**, black). **ECR**: genomic locations of DHS conserved in mouse and rabbit [17], [54], CpG island is indicated by dark green bar. (**B**) Close-up of Wap region from (**A**). **H3Ac**: Summary of H3Ac-ChIP results for Wap promoter and HSS2 (see also Fig. S3) in lactating mammary gland (**Lact**, blue) and Liver tissue (**liver**, red): closed rectangle indicates enrichment of H3Ac at site, open rectangle indicates lack of enrichment at site. **DHS**: Summary of DHS results for rabbit from [17], [54]. Closed rectangle indicates presence of DHS, open rectangle indicates absence of DHS.

**Figure 4:**
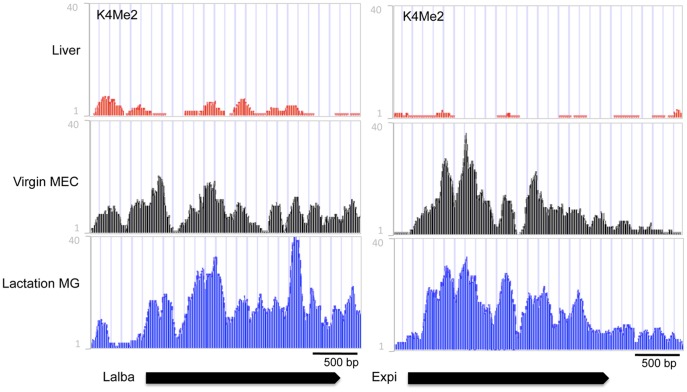
Alpha-lactalbumin (Lalba) and extracellular proteinase inhibitor (Expi) developmental chromatin organization. ChIP-seq reads for H3K4me2 in liver tissue (**Liver**, red), mammary epithelial cells isolated from 12 week virgin mammary glands (**Virgin MEC**, black), and lactating mammary glands (**Lactating MG**, blue). In the bottom panel, black arrows indicate location and transcriptional direction of the Lalba and Expi genes.

To determine the presence of histone modifications associated with open chromatin in milk protein gene loci in mammary and non-mammary tissues, we performed ChIP-seq analysis using an antibody against H3K4me2. H3K4me2 is associated with open chromatin and marks active and poised gene promoters as well as active distal regulatory elements [22]. ChIP-seq showed that the casein gene cluster is enriched for H3K4me2 in lactating mammary gland tissue but lacks this modification in liver tissue (Fig. 1, 2). Neighboring genomic regions containing genes that are expressed in both tissues show enrichment for H3K4me2 in both cases (Fig. 1 and S1A). Conversely, genes expressed in the liver and not in the mammary gland such as the Ugt- and Sult-family, and albumin (Alb)–a liver-specific gene located ∼4 Mb distal to the casein region–as well as tyrosine aminotransferase (Tat) another liver specific gene located on chr 8, show H3K4me2 enrichment in liver (Fig. 1, S1A, B and S2) and not the mammary gland. Meanwhile, the genes immediately flanking the casein gene cluster that are predominantly expressed in salivary gland and tooth development (Smr3a, Smr2, Prol1, Ambn, Enam) but not in mammary gland or liver, lack K4me2 in both these tissues (Fig. 1 and S1A). ChIP-qPCR confirmed H3K4me2 enrichment at the casein gene promoters in the lactating mammary gland compared to liver tissue and at the liver-specific Alb and Tat promoters in liver tissue compared to the lactating mammary gland (Fig. S3A). Furthermore, ChIP-seq showed that genomic regions harboring milk-protein genes such as alpha-lactalbumin (Lalba), extracellular proteinase inhibitor (Expi) and whey acidic protein (Wap) were enriched for H3K4me2 in the lactating mammary gland compared to liver tissue (Fig. 3, Fig. 4, S1C). In summary, these results demonstrate enrichment or lack of H3K4Me2 that is consistent with milk protein gene expression in these tissues.

To further establish the presence of open chromatin on the promoters of milk protein genes and evolutionary conserved regions (ECRs), we used conventional ChIP for Histone H3–acetylation (H3Ac)–associated with open chromatin. H3Ac was enriched on the promoters of the casein genes and Wap, while no enrichment was detected in liver (Fig. 2B,3B, S3B). As expected, the liver-specific albumin promoter showed enrichment for H3Ac in the liver, but not in the lactating mammary gland (Fig. S3B). ChIP analyses also showed that several of the evolutionarily conserved regions (ECRs) analyzed, including BCE, ECR3, ECR19, ECR16 in the casein region and HSS2 in the Wap region are enriched for H3Ac in the lactating mammary gland, but not in the liver (Fig. 2B, 3 and S3B, C). Taken together, milk protein gene promoters and ECRs are enriched with both H3K4me2 and H3Ac, markers of open chromatin, in the lactating mammary gland relative to liver tissue.

#### DNase I hypersensitive site (DHS) mapping shows open chromatin at casein gene promoters and ECRs

We analyzed the Csn1s1, Csn2 and Csn3 gene promoters for DHS in late pregnant (day 16) and lactating mammary gland tissue. We found DHS overlapping the proximal promoter regions of these genes (Fig. 2B and S4A, B, C). The Beta-Casein upstream Enhancer (BCE) region showed three DHSs flanking or within the BCE (Fig. S4B). A strong DHS was located in ECR19 (Fig. S4D). Two DHS flanking ECR6 were previously described for both mouse and bovine lactating mammary gland tissue [35]. No DNase I hypersensitivity was detected at any of these sites in the liver (Fig. 2 and S4A, C, D and data not shown). Consistent with open chromatin during lactation, DHS are present in casein gene promoters and a number of ECRs in the lactating mammary gland, but not in liver tissue.

#### DNA methylation status of casein gene region promoters and distal regulatory elements in lactating mammary gland

**Figure 5:**
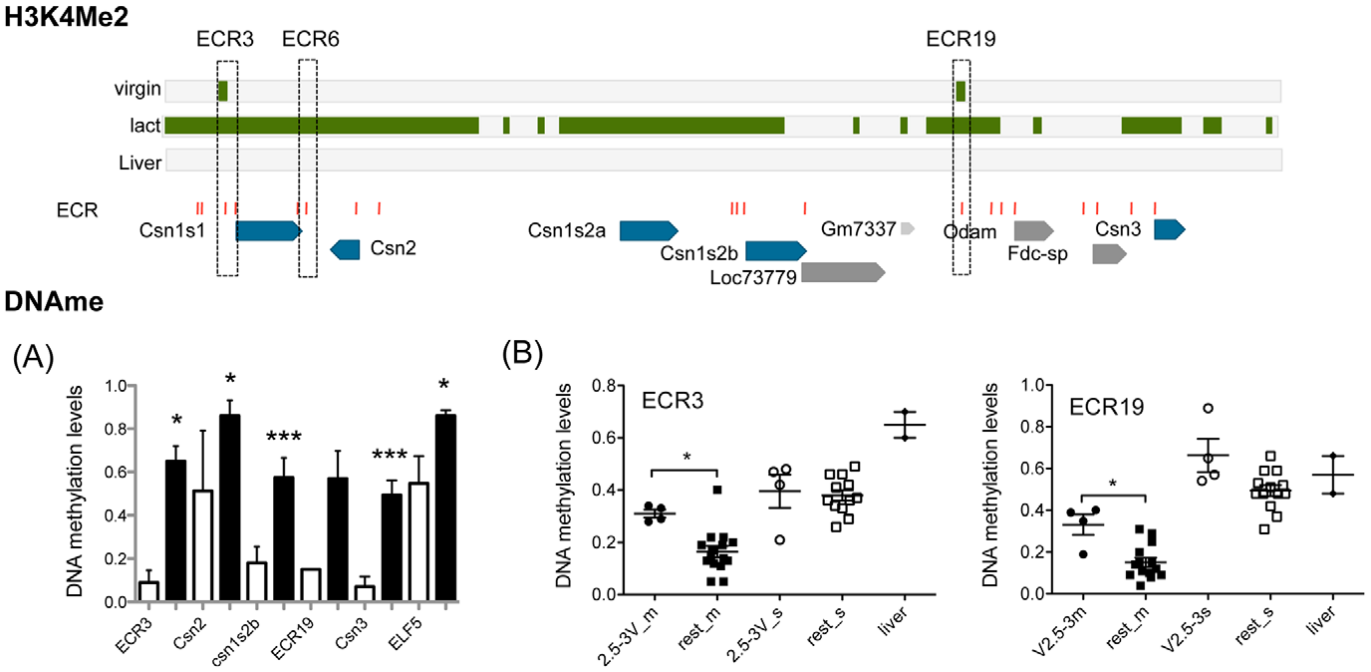
Tissue-specific DNA methylation, and DNA methylation for ECR3 and 19 during mammary gland development. Top panel: Summary of H3K4me2 levels, ECR and gene locations in the CSN region (based on Figure 2). (**A**) Tissue specific DNA methylation levels: DNA methylation levels in lactating mammary gland (white bars) and liver tissue (black bars). Significance was determined using an un-paired, 2-tailed t-test: *<0.05, ***<0.0001. For ECR3 (MG, n = 2; Liver n = 2) and ECR19 (MG: n = 1; Liver n = 2) MeCpG percentages were determined with SEQUENOM mass-array technology (see table S2). Beta-casein promoter (Csn2) (MG, n = 5; Liver n = 5), Alpha-s2b casein promoter (Csn1s2b) (MG, n = 5; Liver n = 5), and Kappa casein promoter (Csn3) (MG, n = 4; Liver n = 4), were determined by bisulfite-sequencing (See Fig. 6 for details). (**B**) DNA methylation levels during mammary gland development and differentiation. MeCpG levels for ECR3 (average of 5 CpG sites in 561 bp) and ECR19 (average of 6 CpG sites in 599 bp) were determined with SEQUENOM mass-array technology (see table S2), different developmental time points are depicted with different symbols, each symbol represents an independent MEC or non-MEC prep from pooled tissue samples (see MEC prep) for virgin samples, 7day pregnant, INV and AMV, or individual animal (16 day pregnant and 8day lactating). *Mammary epithelium (filled symbols*) Pre-pubertal, filled circles (2.5-3V_m: 2.5–3 week old virgin females); Post-pubertal, filled square (rest_m: 3.5–4 week old virgins; 5.5–6 week old virgin females; 8–15 week old virgins; 7day pregnant females; 16 days pregnant; 8 day lactation; Inv (>28 day after lactation); Age Matched Virgin (Virgin animals same age as >28 day involuted animals)**.** *Non-MEC cell fraction of the mammary gland (open symbols*): pre-pubertal: 2.5-3V_s, open circles: 2.5–3 week old virgin females; post-pubertal: rest_s (3.5–4 week old virgin females; 5.5–6 week old virgins; 8–15 Week old virgins; 7day pregnant females; Inv (>28 day after lactation); Age Matched Virgin (Virgin animals same age as >28 day involuted animals). *Liver tissue* **:** filled diamond.

DNA methylation is thought to represent a closed chromatin structure and has been associated with repression of gene expression [27]. The DNA methylation status at the promoters and ECRs in the casein gene region was assessed using bisulfite sequencing, methylation sensitive (HpaII) restriction digest assays, or SEQUENOM mass-array [49], [50] analyses. CpG sites in the promoters and ECRs were methylated at lower levels in lactating tissue than in non-mammary tissues (p<0.05; Fig. 2B, 5A; Table S2). DNA methylation at the Csn2 promoter was ∼40% less in lactating mammary gland than in the liver. DNA methylation at ECR3 was ∼86% less in the mammary gland than the liver. Similar differences were observed for the Csn1s2b and Csn3 promoters (69% and 86% less respectively) and for ECR19 (74% less) (Fig. 2B, 5A; Table S2) and ECR6 (Fig. S5). Consistent with the expression of casein genes in the lactating mammary gland, levels of DNA methylation at casein gene promoters and ECRs are lower in the lactating mammary gland relative to the liver.

Taken together, the above data establish that milk protein genes reside in genomic regions with an open chromatin organization, in the lactating mammary gland but not in non-mammary tissues such as the liver. GAS-(Stat), C/ebp sites and other transcription-factor-binding-sites have been extensively mapped in casein gene promoters and BCE (reviewed in, [15]) and in the WAP promoter and HSS [16], [17]. Consistent with these studies, our data show open chromatin in these regions in the lactating mammary gland.

Is this open chromatin structure a result of chromatin reorganization during development and functional differentiation or is it an intrinsic property of the mammary gland epithelium? We address this question in the remainder of this paper by comparing epigenetic marks across the lactation cycle.

### Progressive Change of Chromatin Marks with Functional Differentiation of the Mammary Gland

The lactating mouse mammary gland consists of >80% epithelial cells while the virgin gland contains a lower percentage of epithelial cells compared to non-epithelial (stromal) cells [51]. To be able to investigate the chromatin organization in mammary epithelial cells (MEC), we prepared organoid preparations that are enriched for MEC [52]. This enabled us to compare the DNA methylation status and H3K4me2 enrichment of the MEC cell population in the mammary gland at different stages of development and functional differentiation.

#### Loss of DNA methylation during functional differentiation

**Figure 6:**
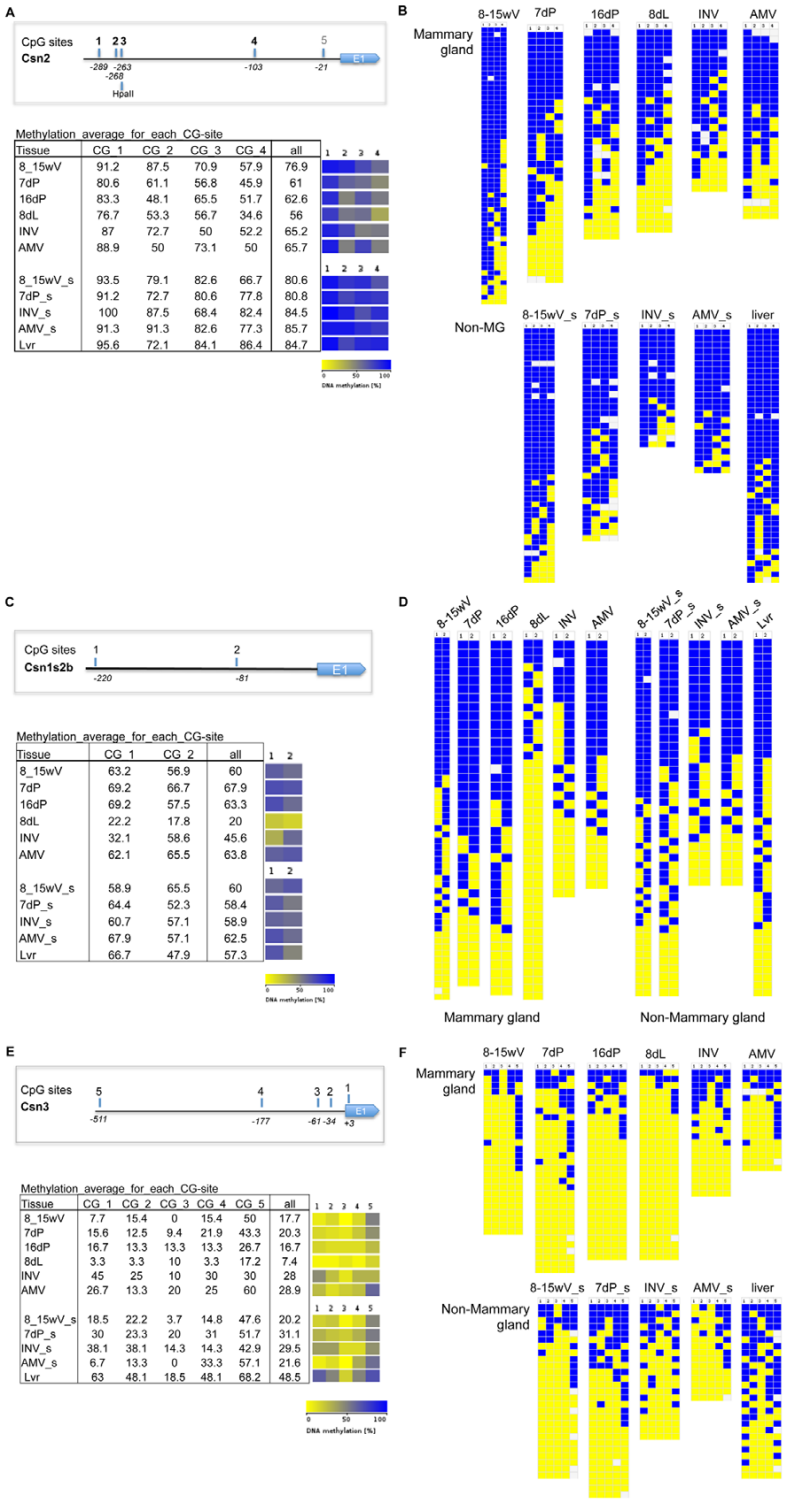
DNA methylation levels during mammary gland development and differentiation at casein gene promoters. (**A, B**) DNA methylation levels (%) for Beta-casein promoter (Csn2) (average of 4 CpG sites in 326 bp), (**C,D**) Alpha s2b casein promoter (Csn1s2b) (average of 2CpG sites in 300 bp) and (**E,F**) Kappa casein promoter *(Csn3)* (average of 5 CpG sites in 588 bp) were determined by bisulfite cloning and sequencing. *Mammary epithelial Cell enriched fractions (MEC)*: Virgin (8_15V; 8–15 week old virgins,); Pregnancy (7P: 7day pregnant females); Inv (>28 day after lactation); AMV: Age Matched Virgin (Virgin animals same age as >28 day involuted animals). *Whole tissue:* pregnancy (16P: 16 days pregnant); Lactation (8L; 8 day lactation). *Non-mammary gland: none-MEC cell fraction of the mammary gland* (8_15; 8–15 Week old virgins); Pregnancy (7P: 7day pregnant females); Inv (>28 day after lactation); AMV: Age Matched Virgin (Virgin animals same age as >28 day involuted animals)); *or non-mammary tissue*: Liver. (**A,C,E**) Overview of Csn2 (**A**), Csn1s2b (**C**) and Csn3 (**E**) promoter and CpG sites analyzed, Tabular representation of DNA methylation levels (%) at individual CpG sites in analyzed region, and DNA methylation levels (%) heatmap representation (yellow 0%-dark blue 100%);. (**B, D, F**) Methylation status at CpG sites in Bisulfite-sequence clones for mammary gland or non-mammary gland tissue (yellow 0% methylation, dark blue 100% methylation): (2–6 individual preps (mammary gland MEC (8-15wV 8–15, 7dP, INV, AMV) or Non-Mammary gland epithelial cells (8-15wV_s, 8-15_s, 7dP_s, INV_s, AMV_s)) and 4–6 individual animals (16dp, 8dL, Lvr), 5–10 clones/DNA prep), Csn2 (C), Csn1s2b (E), Csn3 (F).

DNA methylation levels at ECR3 are similar in MEC and non-MEC from pre-pubertal animals, 2.5–3 weeks of age (Fig. 5B), while they are higher in pre-pubertal MEC compared to mammary gland at all later developmental time points together (Fig. 5B). DNA methylation at ECR19 showed comparable results (Fig. 5B). DNA methylation around ECR6 decreased with pregnancy and lactation (Fig. S5), comparable to the changes detected for the beta-casein promoter (Fig. 6). For the casein promoters (Csn2, Csn1s2b, Csn3), a reduction of DNA methylation levels in pregnancy and/or lactation was detected, (in Csn2 mostly this is reflected by changes at one particular CpG–site 4) (Fig. 6). These changes occur concurrently with the major induction of expression of Csn2 and Csn3 genes in pregnancy and Csn1s2b upon parturition (Fig. S6). Csn2 expression has been detected in virgin mouse tissue, however it is associated with a small subset of cells that undergo secretory differentiation during estrus cycle (and require pregnancy for the establishment of terminal differentiation [53]). The samples we analyzed were staged for estrus cycle to control for differences between samples due to differences in chromatin and expression profiles in cycling animals. Thus, if Csn 2 were to be expressed at the time point studied in the virgin gland this would represent a small number of cells as shown by in situ by Robinson et al. [53], the chromatin organization of such a small subset of cells would not be revealed by the aggregate MEC analysis preformed here. We show that Csn2 expression in virgin MEC is 5 orders of magnitude lower than in the lactating gland (q-RT-PCR, Fig. S6).

As the BCE sequence does not contain CpGs and the proximal promoter sequences (<500 bp from TSS) of Csn1s1 and Csn1s2a did not allow design of working DNA methylation assays for these genes, these regions were not analyzed. However, for all measurable regions, the results are consistent with a loss of DNA methylation near milk protein genes as the mammary gland prepares for lactation.

#### Histone modifications in the mature virgin mammary gland

ChIP-seq analysis showed that, in the casein gene region, levels of H3K4me2 are low in mature virgin MECs compared to lactating mammary gland tissue (Fig. 1, 2 and S3). Whereas, in the neighboring genomic regions containing genes expressed in the mammary gland at all stages, H3K4me2 levels are the same between virgin and lactating MECs (Fig. 1, 2 and S1). Unlike the rest of the casein region, ECR19 showed an H3K4me2 peak in virgin MEC and a small H3K4me2 peak was also detected on ECR3 (Fig. 2). In addition, we detected a DHS at ECR19 in 8 week virgin MEC (Fig. 2 and S4E) and low levels of DNA methylation during/post-puberty (Fig. 5). Taken together, these findings suggest that ECR19 and ECR3 have an open chromatin organization in the virgin gland.

Like the casein region, the Wap gene showed similarly low levels of H3K4me2 in virgin MEC, except for a peak that coincides with HSS0–a DHS identified in mouse, rat (rHSS1) and rabbit lactating mammary gland [17], [38], [54] (Fig. 3B). This HSS0 was shown to persist after lactation in the rabbit [54]. Furthermore this site was shown to be important for high-level lactating mammary gland expression of a rat Wap promoter based transgene. [38], [55], [56]. In contrast to the casein region and the Wap gene, the Lalba and Expi genes are enriched for H3K4Me2 in the virgin gland (but not in liver) (Fig. 4). This implies that different milk protein genes undergo different epigenetic regulation during development and functional differentiation.

**Figure 7:**
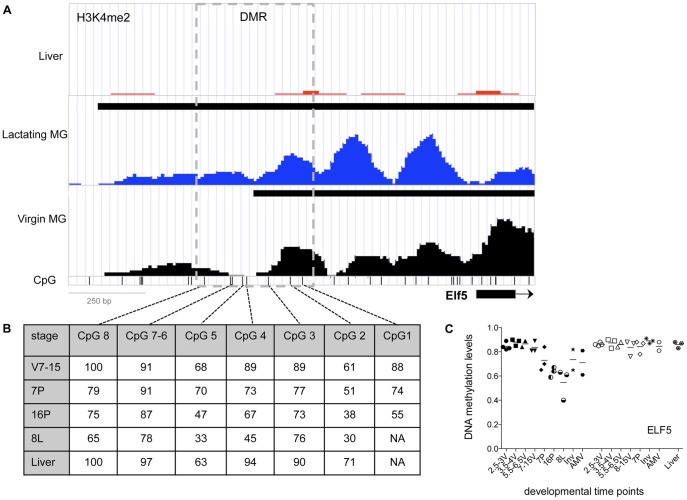
Summary of chromatin organization in the Elf5 region. (**A**) ChIP-seq for H3K4me2 in liver (red), lactating mammary glands (blue) and mammary epithelial cells isolated from 12 week virgin mammary glands (black). Black bars at the top of the Liver, Lactating MG, and Virgin MG panels show the H3K4me2 enriched regions identified by MACS. **CpG**: genomic locations of CpG sites in the 5′ flanking region, exon1 & part of intron 1 (−1418 to +189); DMR indicates the differentially modified region analyzed. (**B**) Table: shows average levels of DNA methylation (%) at developmental stages for individual CpG sites in region analyzed by SEQUENOM mass-array. (**C**) Scatter plot of the changes in DNA methylation levels at different time points during mammary gland development and differentiation at DMR in Elf5 promoter: different developmental time points are depicted with different filled symbols. *Mammary epithelial cell enriched fractions (MEC)*, filled symbols: Virgin (2.5_3V: 2.5–3 week old virgin female, filled circle; 3.5–4V, 3.5–4 week old virgin, filled square; 5.5_6V: 5.5–6 week old female virgin, filled downward pointing triangle; 8_15V; 8–15 week old virgin, filled upward pointing triangle); Pregnancy (7P: 7day pregnant, filled diamond; 16P: 16 days pregnant (whole tissue), half vertically filled circle); Lactation (8L; 8 day lactation (whole tissue), half horizontally filled circle); Inv (>28 day after lactation) 5-pointed star; AMV: Age Matched Virgin (Virgin animal same age as >28 day involuted animal), filled hexagonal. *Non-MEC cell fraction of the mammary gland* (Non-MEC, open symbols): 2.5_3: 2.5–3 week old virgin female, open circle; 3.5–4V, 3.5–4 week old virgin, open square; 5.5_6: 5.5–6 week old female virgin, open downward pointing triangle; 8_15; 8–15 Week virgin, open upward pointing triangle; Pregnancy (7P: 7day pregnant, open diamond); Inv (>28 day after lactation, 8 pointed star); AMV: Age Matched Virgin (Virgin animal same age as >28 day involuted animal), open hexagonal); or *Non-mammary tissue* (hexagonal with star): Liver.

ELF5 is an important regulator of functional differentiation during pregnancy, regulating the establishment of the secretory alveolar lineage and expression of some milk protein genes [57]–[59]. In the ELF5 promoter, H3K4me2 enrichment is present in both the virgin and lactating epithelium, but the enrichment extends further from the transcription start site in the lactating mammary gland compared to virgin MECs. The boundary of the enriched region in the virgin MEC coincides with the region that is differentially methylated during development [59] (Fig. 7).

**Figure 8:**
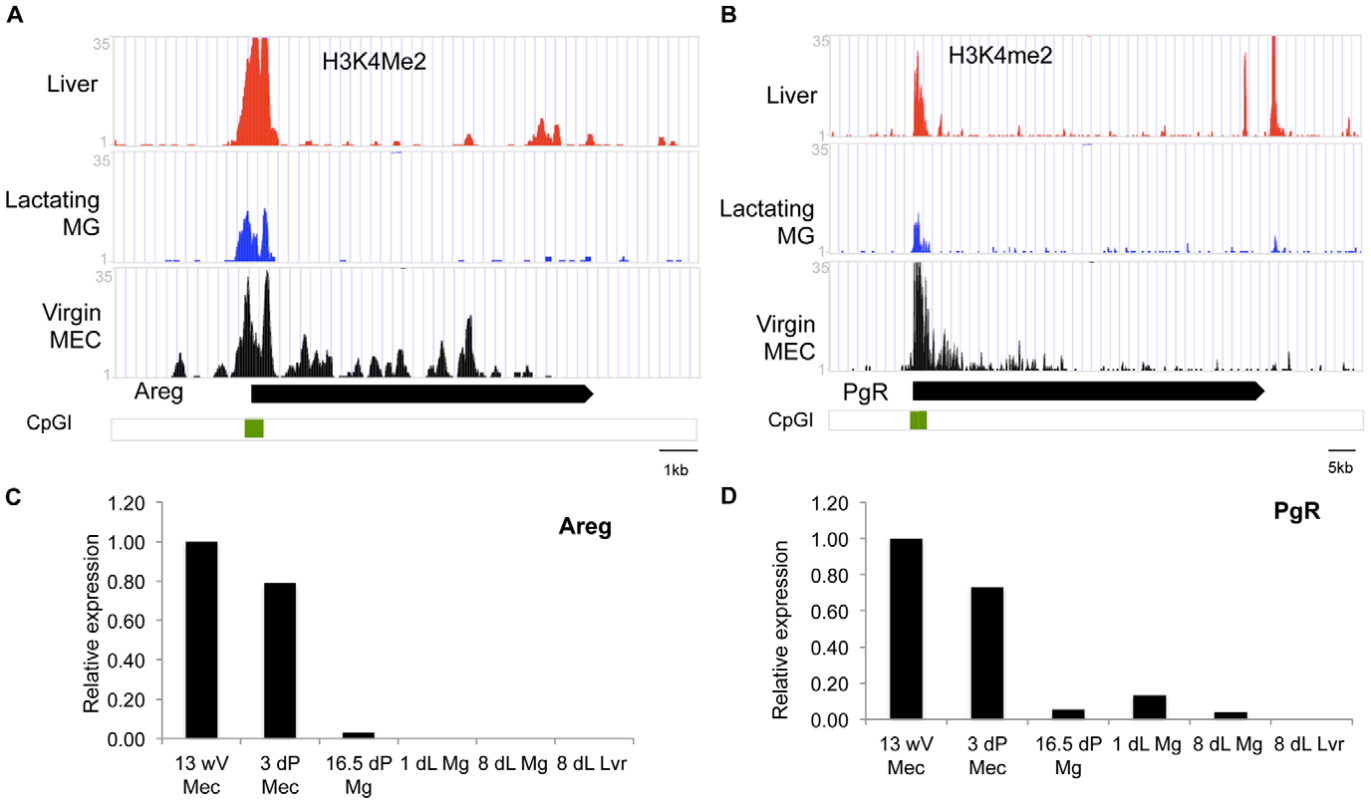
H3K4me2 ChIP-seq and RT-PCR results for genes expressed in virgin mammary gland. (**A–B**): ChIP-seq reads for H3K4me2 in liver tissue (**Liver**, red), lactating mammary glands (**Lactating MG**, blue) and mammary epithelial cells isolated from 12 week virgin mammary glands (**Virgin MG**, black). (**C–D**): Q-RT-PCR of (**C**) Amphiregulin (Areg) and (**D**) Progesterone receptor PgR at different developmental stages in MEC isolated from mammary gland tissue of 13 week virgin (13wV), 3 days pregnant (3dP) or whole mammary tissue from 16.5 day pregnant (16.dP Mg) day 1 and day8 lactation (1dL Mg and 8dL MG) as well as liver from an 8d lactating animal (8dL Lvr). Real-time-RT-PCR data are normalized to Keratine 18 and expressed relative to 13wV MEC.

Consistent with the fact that the amphiregulin (Areg) and progesterone receptor (Pgr) genes are mainly expressed in the virgin MEC, H3K4me2 was markedly enriched on these genes in the virgin MEC (Fig. 8). In the lactating mammary gland and the liver, in which Areg and PgR are not expressed, H3K4me2 was only present on the promoter CpG islands of these genes (Fig. 8). The presence of H3K4me2 on CpG islands has been suggested to protect them from DNA methylation [60]. DNA in CpG islands is generally unmethylated in normal tissues irrespective of the transcriptional status of the gene with which they are associated. In accordance with this, we detected low levels or no CpG methylation on the Areg promoter in all 3 tissues regardless of expression (data not shown).

Together, these results indicate that chromatin organization gradually changes with the development and functional differentiation of the mammary gland and is associated with changes in gene expression.

#### Involution

Cessation of lactation results in involution of the mammary gland, which is accompanied by a profound reorganization of the mammary gland. Many of the epithelial cells that produced milk undergo apoptosis, while the adipocytes reconstitute their lipid content [5]. DNA methylation levels of the Csn2, Csn1s2b and Csn3 promoters in MEC isolated from involuted (>28 days) mammary tissue were on average higher than in lactation and comparable to non-MEC and to MEC isolated from Age Matched Virgins (AMV) (Fig. 6). In contrast, the methylation levels of ECR3 and 19, which were already low at the start of functional differentiation (pregnancy, lactation), remained low after involution (Table S2). These results suggest that the milk protein gene loci may return to a silenced state during involution. However, the great variation in methylation levels of individual samples within time point prevents firm conclusions and more extensive studies on the chromatin state of the involuted gland are needed.

## Discussion

### Spatial and Temporal Epigenetic Regulation of Milk Protein Genes

DNAseI hypersensitivity (DHS), histone H3 acetylation (H3Ac) enrichment, and H3K4-di-methylation (H3K4me2) enrichment are each indicative of open chromatin [25], [61]. The presence of these open chromatin marks at casein promoters and ECRs in lactation indicates that these regions have an open chromatin structure in the functionally differentiated gland. In addition, DNA methylation levels–associated with closed chromatin–in these regions are lower compared to non-mammary tissue. Together, these data suggest that the casein gene region has an open chromatin configuration in the lactating mammary gland.

In contrast, in liver tissue the casein gene cluster region lacks H3K4me2 and has higher levels of DNA methylation relative to mammary gland tissue, both of which are indicative of closed chromatin structure. Furthermore, our preliminary data show enrichment for H3K27me3–another histone modification associated with developmentally inactivated chromatin–in the casein locus in liver tissue (data not shown). These findings are consistent with data from Wen et al [62] in which the casein gene region is encompassed in an H3K9me2 “LOCK”–a region of closed chromatin–in mouse liver (Fig. 2B). The same chromatin organization, open in lactating mammary gland tissue and closed in liver, was detected for other milk protein genes. Thus, regulatory regions of the milk protein genes are characterized by an open chromatin structure in the lactating mammary gland, but by closed chromatin in other tissues, demonstrating tissue-specific epigenetic regulation of these milk proteins.

A lack of H3K4me2 enrichment at the promoters and throughout most of the casein gene region in the virgin MEC, together with a trend towards higher levels of DNA methylation in virgin MEC, suggest a more closed chromatin organization relative to lactation in the mature virgin gland. However some ECRs (ECR3 and ECR19) show a reduction of DNA methylation from pre- to post-puberty and an open chromatin organization marked by H3K4Me2 enrichment in the mature virgin, suggesting an early role for these ECR in gene regulation in the casein region. Similar changes in chromatin organization between the virgin and lactating mammary gland were seen in the WAP gene region–Hss0 is enriched for H3K4me3 in the virgin gland. These epigenetic changes are consistent with the high level of expression of these genes during lactation.

In contrast to the casein and Wap gene regions, H3K4me2 enrichment of the Lalba and Expi gene regions in the mature virgin gland indicates that these regions already have an open chromatin organization at this stage of development. These seemingly contradictory results highlight the fact that gene expression is the summation of different mechanisms of regulation. This suggests that these genes are regulated differently and that an open chromatin organization at these genes is not enough for gene expression. Interestingly in a study examining the role of Sim2s in mammary gland function Wellberg et al [63] show that over-expression of Sim2s in the mouse mammary gland results in enhanced Csn2 and Wap gene expression while there is no effect on Lalba and Expi. Taken together this suggests that the differences in chromatin organization in the virgin gland are a reflection of the differential transcriptional regulation of these genes. On the other hand, the Areg and Prg genes, expressed in the virgin gland and not expressed or at much lower levels during lactation (or in liver tissue), have very little or no H3K4me2 on the gene in lactating mammary tissue (and in liver tissue) while they are enriched in the virgin gland.

After involution of the mammary gland, a time when casein genes are not expressed at high levels, DNA methylation levels at the Csn1s2b casein gene promoters are higher than during lactation and pregnancy and a similar trend was present for the Csn2 and Csn3 promoters, whereas the DNA methylation levels at ECR3 and 19 remain low. This suggests a more closed chromatin state at the promoters in the involuted gland while the chromatin organization at potential DRE (ECR3 and 19) appears to remains open.

Together, these data indicate that epigenetic modifications influence temporal regulation of gene expression in the mammary gland. Moreover, our results suggest that there are distinct groups of genes, each with their own dynamic pattern of epigenetic regulation during mammary gland development.

### Epigenetics in Involution of the Mammary Gland

Upon involution, milk protein gene expression is down-regulated, tissue remodeling takes place and morphologically the gland resembles the pre-pregnant state. The transcriptional state of the mammary gland is at least partially reset to the level prior to the onset of lactation [11]. This observation is consistent with our DNA methylation results. However, some changes in mammary gene expression persist [64]–[67] including a ∼2 fold higher levels of Csn1s1 and Csn2 expression in the parous vs nulli-parous non-lactating mammary gland (J. Jerry and M. Hagen personal communication). It has been suggested that the gland is altered at the molecular level including chromatin changes in MECs that persist after pregnancy and lactation [68].

Higher DNA methylation levels at the casein gene promoters after involution than during lactation and pregnancy (Fig. 6) could be due to two, not necessarily mutually exclusive, mechanisms: 1) the promoters become remethylated by de-novo methylation when gene transcription ceases (as suggested by results for the bovine ECR3 [69], [70]), 2) the cells that have undergone the terminal differentiation step involving demethylation of these promoters are lost during the massive apoptosis and remodeling that occurs upon involution. The latter does occur, but it is not clear if only these cells have low methylation levels at the promoters. Analysis of different chromatin modifications in MEC of parous vs. nulliparous animals is underway and should provide further insight into this question of persistence of chromatin changes after lactation and their relation to persistence of physiological changes.

### Role of Distal Regulatory Elements (DRE) in Spatial and Temporal Gene Regulation

Studies of DREs in different tissues and in other gene regions such as beta-globin [71] T-helper 2 cytokine, Ifng, and Il17a–Il17f locus during T-helper-cell differentiation [72], the human Growth Hormone cluster [73] and H19/Igfr2 [34] have demonstrated that DREs can establish a chromatin structure favorable for further chromatin remodeling during differentiation and lineage commitment. Evolutionary conserved regions ECR3 and ECR19, which are distal to the casein gene promoters, may have a similarly function as DREs. Our results demonstrate that epigenetic modulation of ECR3 and ECR19 occurs much earlier than at the casein promoter and gene regions itself. These epigenetic modifications at ECR3 and ECR19 are both spatially and temporarily specific: histone H3 hyper-acetylation, enrichment for H3K4me2 and DNase I hypersensitivity (DHS) in the lactating mammary gland as compared to the liver (and non-MEC), DHS and H3K4me2 enrichment in virgin MEC relative to lactation, and a trend of decreasing DNA methylation in MEC during pubertal development. Importantly, the open chromatin organization on ECR3 and 19 is attained during puberty as suggested by DNA methylation analysis. In other words, we have observed open chromatin structure at ECRs in the vicinity of milk protein genes, before these genes have reached their full expression potential. Therefore, our data are supportive of the hypothesis that the casein locus is regulated by DRE [35], [36], and we hypothesize that ECR3 and ECR19 are DREs, which establish a chromatin structure that enables further chromatin remodeling and changes in 3D chromatin interactions, which lead to casein gene expression.

Transcription factor binding site predictions as well as genomic location suggest that ECR19 may serve as a chromatin-organizing element. ECR19 and a 1 kb region immediately flanking have predicted binding sites for the chromatin organizer CTCF [74] (http://insulatordb.uthsc.edu/ [75]). CTCF is a DNA binding factor shown to bind DNA elements that function as chromatin organizer (e.g. insulators, boundary elements, chromatin-loop anchor) and is often located at the borders of chromatin domains [74], [76]. CTCF-ChIP analyses from the ENCODE data set indicate that CTCF binds the immediately flanking region in several human and mouse non-mammary cell types ([77]–[79] and http://genome.ucsc.edu/). ECR19 is located between the region harboring four of the casein genes, which are expressed in the mammary gland, and the Odam gene, which is expressed in tooth-associated and other epithelia [80]. In summary, the location of ECR19, its transcription factor binding sites predictions, and its epigenetic modifications leads us to hypothesize that ECR19 organizes the chromatin between genes expressed in different types of epithelial tissue, and its activation during functional differentiation of the mammary gland could help prevent expression of non-mammary genes during this highly active stage. Targeted experimentation should be conducted to confirm such a role.

Our findings should be interpreted with the following caveats. First, the virgin MEC preparations are not 100% pure mammary epithelial cells, and likewise neither does lactating mammary tissue consist of 100% epithelial cells, but rather both are (significantly) enriched for MECs. Second, two recent studies demonstrated lineage specific (stem/progenitor/basal vs. luminal) epigenetic marks in mammary epithelial cells [48], [59]. Third, there are differences in the presence of ductal luminal epithelial cells and alveolar luminal epithelial cells between the two developmental stages studied. These limitations might have implications for the interpretation of our results. Nevertheless, it is clear that there are distinct epigenetic states in the cell types that prevail at each developmental stage. To what effect they represent a continuum of epigenetic states in the same cell population during development will require further study.

We have intensely studied a small subset of genes, particularly the casein genes, so there are limits to which these findings can apply to the whole genome. However, similar findings for other spatially, temporally and lineages regulated genes in different tissues [34], [71]–[73] show the general applicability of these studies and the mammary gland is an excellent model system for the understanding of the role of epigenetic regulation in development and functional differentiation of somatic tissue. In contrast to whole genome studies, this focused study of the casein region enabled us to finely characterize regions of interest such as the evolutionary conserved regions and learn more about the regulation of casein gene cluster, encoding major milk proteins, with major nutritional functions. Lessons learned here can be applied in future studies at a genome-wide scale.

## Materials and Methods

### Animals/ethics Statement

Balb/c and ICR mice were obtained from Harlan laboratories and housed in an American Association of Laboratory Animal Care-accredited facility at Baylor College of Medicine following guidelines outlined by the institutional Animal Care and Use Committee. The protocol was approved by institutional Animal Care and Use Committee of Baylor College of Medicine (AN-3455). Animals were euthanized by CO_2_ exposure and tissues were isolated from animals at different ages and times of mammary gland development.

### Epithelial Cell Enrichment

Mammary Epithelial Cell (MEC) enriched organoid preparations were obtained by enzymatic digestion of mammary gland tissue and differential sedimentation as described by [52]. In short, #3,4 and 5 glands were harvested from 12 week old virgin animals (staged for estrous cycle at diestrus: 10–15 animals per prep), and minced with razor blades, enzymatically dissociated with 0.2% collagenase A (Roche) and 0.2% trypsin (Gibco) for 30 min at 37°C with shaking. Organoids were sedimented at 450 g and washed twice in F12 media. Single cells were depleted by sedimentation of the organoid by repeated short pulse spins at 450 g. The supernatant of the second wash and the first pulse spin were collected as non-MEC cell fraction (mainly fibroblasts). MEC enriched organoids and the non-MEC fraction were used for DNA isolation and/or DNaseI hypersensitive site mapping. MEC preps for other developmental time points we prepared from pooled tissue from: 20–25 animals for 2.5 to 4 week old animals; 6–15 for animals between 5–15 weeks and involuted as well as age-matched virgins; 4–6 animals were used per MEC prep for early pregnancy time points.

### DNA Isolation

Genomic DNA was isolated from MEC and whole tissues using standard proteinase K and phenol/chloroform methods [81]. DNA concentration was determined with ND-900 Nanodrop spectrophotometer or Picogreen (Invitrogen) measured on Q-bit and Quality was assessed by gel electrophoresis.

### mRNA Isolation and Analysis

Total RNA was isolated from whole tissue (16d pregnant, 8day lactating, liver) or MEC pellets (13week virgin, 3day pregnant) using Trizol (Invitrogen) and treated with DNaseI followed by clean-up using RNAeasy kit (Qiagen). RNA concentration was measured with Ribogreen (Invitrogen) measured on Q-bit and quality was determined using Agilent Bioanalyzer. mRNA was converted into cDNA using SuperScript III reverse transcriptase according to manufacturer’s protocol (Invitrogen). Relative expression was determined using CyberGreen (seeTable S1 for primers) and 2^−ΔΔCt^ method [82]. Values were normalized with Keratin(Krt) 18 (epithelial specific marker) and expressed relative to the 13 week virgin levels.

### Chromatin Immunoprecipitation (ChIP)

Snap frozen lactating mammary gland and liver tissue were crushed with a mortar and pestle under liquid nitrogen, transferred to 1% formaldehyde in PBS and fixed at room temperature for 10 min. Fixation was stopped with 2 M glycine added to a final concentration of 125 mM. Cells were washed 3× in 1X PBS with protease inhibitors (Complete Mini tab, Roche). Next, cells were incubated in Nuclear Isolation Buffer (NEBA: 10 mM Hepes pH 7.9, 10 mM KCL, 0.1 mM EDTA, 0.1 mM EGTA, 0.5% NP40, plus 10 mM Na Butyrate and protease inhibitors (complete mini tab, Roche)) on ice for 20 min. Nuclei were released from cells by Dounce homogenization (loose pestle) with 10–15 strokes. Nuclei were collected by centrifugation at 500 g at 4°C for 5 min. Nuclei were lysed in nuclear lysis buffer (NLB: 50 mM Tris pH 8.0, 10 mM EDTA, 1% SDS plus 10 mM Na Butyrate and protease inhibitors (Complete Mini Tab, Roche)) and chromatin was sheered to a size of 200–1000 bp by sonication (Branson D-450) using 15 sec bursts (total of 1.45 min), 30 sec rests, at 90 to 100% Amplitude. 50–150 µg of chromatin was used per Immunoprecipitation (IP) in 1× dilution of ChIP Dilution Buffer (IPDB (10×): 16.7 mM Tris-Cl pH 8.1, 167 mM NaCl, 1.2 mM EDTA, 1.1% Trition X100, 0.01% SDS, plus 10 mM Na Butyrate, 10 mM PMSF and protease inhibitors (Complete Mini tab, Roche)). Chromatin was precleared with protein-A Sepharose beads (16–157, Upstate-Millipore) and normal rabbit serum (Sigma Cat# 15006) for 1 hr at 4°C. Chromatin was incubated with 5 µg antibody specific for H3Ac (06–599. Upstate-Millipore); H3K4me2 (07–030, Upstate-Millipore); Pol II (sc-899X, Santa Cruz) (Table S3) overnight at 4°C with top over rotation The next day protein-A Sepharose beads were added for 1 hr under rotation at 4°C to collect antibody bound chromatin. Bound chromatin was washed with Low Salt Wash Buffer (0.1% SDS, 1% Triton X100, 2 mM EDTA, 20 mM Tris pH 8.1, 150 mM NaCl), High Salt Wash Buffer (0.1% SDS, 1% TX100, 2 mM EDTA, 20 mM Tris pH 8.1, 500 mM NaCl), Lithium Chloride Wash Buffer (250 mM LiCl, 1% NP40, 1% Deoxycholate, 1 mM EDTA, 10 mM Tris pH 8), and TE 1(0 mM Tris pH 8.0, 1 mM EDTA pH 8.0) under rotation at 4°C for 5 min each, and collected by centrifugation at 3000 g, 1 min at 4°C, eluted from beads with elution buffer (EB: 1%SDS, 100 mM NaCO3, dH2O), and decrosslinked (over night at 65°C). DNA was isolated using PCR columns, (Qiagen). Conventional PCR or real-time-PCR was performed to determine enrichment. 5 µl of input (diluted 50× in 100 ng/µl tRNA) and 5 µl of IP (diluted 5× in 100 ng/µl tRNA) were amplified with primers specific for amplicons covering the ECRs and casein promoters (sequences and PCR conditions (Table S1).

Real-Time-PCR, values were calculated relative to a standard curve of genomic input DNA and were normalized to GAPDH values in mammary gland or liver.

### ChIP-seq Analysis

For ChIP-seq, chromatin was prepared as described by Wagschal et al [83] from MEC preps obtained from 10–15 12-week old virgin animals (staged for estrous cycle at diestrus), and pooled mammary gland or liver tissue from 4–6 mice at lactation day 8. Chromatin IP was performed as described above, deep sequencing libraries were prepared according to illumina ChIP-seq sample preparation protocol and sequenced.

Raw reads generated from Illumina/Solexa GAII were mapped to mouse reference genome (NCBI37/mm9) using Eland (Illumina) with maximally 2 mismatches tolerated.

ChIP-seq data (fig. S7) have been deposited in NCBI’s GEO database: GSE25105. Technical replicates were pooled together to achieve better coverage. Peak calling was performed by MACS (version 1.3.6) and the SICER (spatial clustering approach for the identification of ChIP-enriched regions) algorithm [84] using uniquely aligned reads with input/IgG as background control [85]. The MACS algorithm [85] was used to identify peak regions over smaller well-defined regions such as ECRs. To identify peaks of more diffuse histone modification signals over larger regions, we applied the SICER algorithm [84] to the genome-wide raw sequence reads of H3K4me2 occupation sites in the lactating mammary gland, in virgin MECs, and in liver. Input–seq read libraries were used as a control in both analyses. SICER's default parameters were used except for the change of species to mm9 and the gap size. The window size was kept at 200 bp because this is approximately the length of a nucleosome plus linker. The gap size parameter is a multiple of the window size, but the optimal choice of this parameter depends on the characteristics of the chromatin modification. To determine an appropriate gap size, SICER was iteratively run with increasing gap size and the aggregate island score was plotted as a function of gap multiple to find the gap size for which the maximum is reached. An optimal gap size of twice the window size (gap size = 400 bp) in lactating mammary was chosen and this gap size choice appears to be robust to tissue type (mammary or liver) or the control library. The same procedure was repeated for K4Me2 in virgin MECs, yielding a gap size of three times the window size (gap size = 600 bp).

### DNaseI Hypersensitive Site Mapping

DHS mapping was performed as described by Millot et al [17]. In short: Nuclei were isolated from MEC preps or crushed frozen tissue by cell lysis in A+/NT/L (120 mM Sucrose, 24 mM KCl, 12.5 mM NaCl, 0.8 mM EDTA, 0.4 mM EGTA, 0.04%TritonX-100, 10 mM Tris-HCl pH 7.9, 0.06 mM Spermidine, 0.4 mM Spermine, 5.6 mM beta-Mercaptoethanol, 0.04 mM PMSF, 0.4 mM DTT, 0.05% NP40). Nuclei were then collected by centrifugation and resuspended in A+/NT (300 mM Sucrose, 60 mM KCl, 15 mM NaCl, 2 mM EDTA, 2 mM EGTA, 0.1%TritonX-100, 10 mM Tris-HCl pH 7.9, 0.15 mM Spermidine, 1 mM Spermine, 14 mM beta-Mercaptoethanol, 0.1 mM PMSF, 1 mM DTT), Nuclei were collected by centrifugation and digested in Nuclear Digestion Buffer buffer (NDB: Sucrose 300 mM, 60 mM KCl, 15 mM NaCL, 2 mM EGTA, 2 mM EDTA, 15 mM Tris pH 7.4, 5 mMMgCl_2_, 0.1 mM PMSF, 0.5 mM DTT) with MgCl_2_ and CaCl_2_ added to 0.4 mM and 2 mM respectively at a standard DNA: DNaseI (RQ1 RNase-Free DNase, Promega) concentration for increasing time. The reaction was stopped with 20 mM EDTA, 1% SDS. DNA was isolated with proteinase K treatment and purified by phenol/chloroform extraction. DHS were detected by Southern blotting using probes designed to the ends of informative restriction fragments, or by Real-Time-PCR as described by McArthur et al [86] using primers on each side of HSS. Fragments from the albumin locus or Beta-globin gene were used as non-HSS controls in mammary gland tissue.

### DNA Methylation Analysis

Up to 1 µg genomic DNA was treated with Bisulfite (BS) using the Epitech Bisulfite kit according to manufacturer’s instructions (Qiagen). PCR primers specific for Bisulfite treated DNA were designed with methyl-primer express software (ABI). PCR was performed using Bisulfite DNA specific primers (sequence supplemental data Table S1), 2–4 µl Bisulfite treated DNA, 1× PCR buffer (Invitrogen), 2.5 mM MgCl2, 0.2 mM dNTPs, 0.8 mM primers, and 0.02 U Platinum Taq (Invirogen): 3 min 95°C; 5 times: 1 min 95°C, 2 min 60°C; 3 min 72°C; and 25 times: 1 min 95°C, 1 min 60°C, 1.5 min 72°C; followed by 4 min 72°C and 4°C hold. BS-PCR products were cleaned up by PCR cleanup (Qiagen) or Gel isolation kit (Qiagen) for reactions that demonstrated more than one amplification product to isolate the product of the correct size. For initial differential DNA methylation assessment BS-PCR products were sequenced at the Children’s Health Research Center (CHRC) molecular biology core or LonestarSeq, Houston TX. To determine percentage of methylation in select regions, the BS-PCR product was cloned into pGemT easy vector (Promega), at least 8–10 individual clones were isolated (Qiagen mini prep kit), and sequenced at CHRC molecular biology core or Agencourt bioscience Corp., bisulfite-sequences were processed using BISMA [87] for Csn2, Csn1s2b, Csn3. For ECR3, ECR19 and Elf5, levels of DNA methylation were determined using SEQUENOM’s MassARRAY platform by SEQUENOM Inc. This system utilizes MALDI-TOF mass spectrometry in combination with RNA base specific cleavage (MassCLEAVE) [49], [50] (primers Table S1). DNA methylation in ECR6 was analyzed with DNA methylation sensitive restriction enzyme digests (HpaII/MspI) and Southern blotting.

### Computational Analysis of ECR 19

Sequence was retrieved from available genome assembly. Sequence was analyzed for potential CTCF binding sites at the CTCF binding site data base ([75],http://insulatordb.uthsc.edu/).

### Conclusion

These studies demonstrate that milk protein gene genomic regions have tissue- and developmental-stage specific chromatin organization, which changes with development and functional differentiation of the gland. Furthermore, there are subsets of genes with different epigenetic regulation; for the casein genes and Wap potential distal regulatory elements have an open organization in mature virgin, while chromatin at promoters opens up concomitantly with major induction of gene transcription during pregnancy. For genes like Lalba, Epxi and Elf5, the genes and most of the promoter have already attained an open chromatin structure in virgin tissue.

Collectively, these data show mammary gland development-specific epigenetic marks on regulatory elements of mammary gland-specific genes, representing the progression to an open chromatin structure in a fully functional gland. The progressive gain of epigenetic marks representing open/active chromatin with functional development of the mammary gland suggests a model that sets up a poised chromatin organization during pubertal development that is ready to respond to the signals of pregnancy and lactation to achieve full functional capacity. The observed chromatin changes during mammary gland development and functional differentiation imply that the hormonally-induced signaling pathways of development work in concert with chromatin remodeling and epigenetic regulation to control gene expression and tissue development. These observations also imply that external exposures affecting epigenetic marks and chromatin organization are likely to contribute to altered functioning of the mammary gland. Integration of chromatin status (epigenetics), signaling pathways, gene expression and structural variation of the genome (SNP, CNV) will lead to a better understanding of the functioning of the mammary gland in health and disease, and may contribute to new strategies for enhancement of lactation performance and breast cancer treatment.

Broadly, our data demonstrate chromatin remodeling in both a tissue-specific and developmental stage-specific manner. These data also suggest that the evolutionary conservation of certain sequences in promoters and distal regions is due to their role in regional gene expression through control of chromatin state. Employing the mammary gland as a model for developmental biology, we expect these findings to be widely applicable to the development of other tissues and organs in mammals and in other organisms.

## Supporting Information

Figure S1 **Gene expression of genes in genomic region as shown in** **figure 1** **–** **4** **and S2.** Expression of genes in the casein gene region **(A),** Albumin (ALB) and tyrosine aminotransferase (Tat) gene **(B)**, and other milk protein gens: genes in the Whey Acidic Protein gene region, alpha-lactalbumin (Lalba), and extracellular proteinase inhibitor (Expi) **(C)**, in liver (red) and lactating mammary gland (blue). Expression array data (GDS1805) for liver (GSM96229:, GSM96230, GSM96231) and lactating mammary gland (GSM96203, GSM96204, GSM96205) [88] were retrieved for genes in CSN and Wap regions shown in Fig. 1–4 and S2, mean values of the 3 replicates +SD were plotted in order of location of genes in genomic region as shown in figure 1–4 and S2.
(PDF)

Figure S2 **H3K4me2** **ChIP-seq results for Albumin (Alb) and tyrosine aminotransferase (Tat) gene expressed in liver.** ChIP-seq H3K4me2 of liver (red) lactating mammary gland (blue) and virgin MEC (black) tissue. Black arrow indicates location and transcriptional direction of Alb and Tat, Green box indicates Albumin enhancer location.
(PDF)

Figure S3 **ChIP on select region in the casein gene cluster, Wap Alb and Tat genes.** ChIP assays on mouse lactating mammary gland (white bars) and liver (Black bars) tissue. MG and liver chromatin was immuno-precipitated using antibodies against-acetylated-Histone-H3 and di-methylated-Lysine4 of H3 (H3K4me2), DNA was isolated, and DNA samples were analyzed using real-time PCR. (**A**) enrichment in samples immuno-precipitated with antibodies to (A) H3K4me2 (Csn2 n = 4; Tat n = 4; Alb n = 3; Csn3 n = 2, Csn1s1, Csn1s2a and Csn1s2b n = 1)). (**B**) H3Ac (Csn2, Csn3 and Alb n = 4; Csn2s1, Csn1s2a and Csn1s2b n = 3; ECR3, BCE, Wap and Wap_hss2 n = 2), compared to input (un-precipitated sample) and normalized to enrichment in the housekeeping gene GAPDH, based on real-time PCR analysis of the indicated amplicons.(P values based on 2-sample t-test are indicated; *<0.05; **<0.001; ***<0.0001) (**C**), representative PCR results (n = 3) of ChIP analysis of ECRs (No_Ab: no antibody control; H3Ac: anti-acetylated histone H3 antibody). Alb: albumin gene & Tat; tyrosine aminotransferase (both liver specific); Csn1s1: alpha-s1casein; Csn2: beta casein: Csn1s2a: alpha-s2a casein; Csn1s2b: alpha-s2b casein; Csn3: kappa casein; Wap-p: Whey Acid Protein promoter; Wap-H2: Wap-Hypersensitive site 2; ECR1: evolutionary conserved region 1; ECR3; ECR6; beta-casein upstream enhancer (BCE); ECR16, ECR19, ECR21 (evolutionary conserved regions).
(PDF)

Figure S4 **Tissue-specific DHS on casein promoters and ECRs (BCE, ECR19).** **(A)** Csn1s1 (Alpha casein) promoter: Nuclei were isolated from 16day pregnant and liver tissue, exposed to increasing amounts of DNase1, genomic DNA isolated and digested with EcoRI. Regions in the chromatin hypersensitive to DNase1 are detected as sub-bands of the EcoRI band (red line and red arrow), size of the sub-bands indicates the location of a DNase1 hypersensitive site (vertical arrow head). Red blocks indicate location of probe used. **(B)** Nuclei were isolated from 16day pregnant and8 day lactating mammary gland tissue, exposed to increasing amounts of DNase1, genomic DNA isolated and digested with EcoRI. Analyzed with a probe identifying DHS at the CSn2 (beta casein) promoter (left panel) or DHS around the BCE (right panel). **(C)** Csn3 (kappa-casein) Nuclei were isolated from 16day pregnant and liver tissue, exposed to increasing amounts of DNase1, genomic DNA isolated and digested with EcoRI. **(D)** ECR19: Nuclei were isolated from 16day pregnant and liver tissue, exposed to increasing amounts of DNase1, genomic DNA isolated and digested with EcoRI. Similarly Nuclei were isolated from MEC and non-MEC cell preparations isolated from mammary glands of 8 week old virgin animals, exposed to increasing amounts of DNase1, genomic DNA isolated, PCR performed with primers flanking the HS in ECR19. Values were normalized to fragment that is not DHS in these tissues; DHS is expressed as fraction of fragments left relative to non-MEC and zero point.
(PDF)

Figure S5 **DNA methylation around ECR6 during mammary gland development and in Liver.** Genomic DNA isolated from 3 week virgin (3V) mammary gland, 12 week virgin MEC (12Ve) or non-MEC (12Vs) cells, 8day lactating mammary gland and Liver was digested with EcoRI alone (lanes 3) or in combination with either MspI (lanes 1) or methylation sensitive HpaII (lane2). ECR6 sequence was used as probe in southern blot analysis. Complete digestion with MspI or HpaII results in the detection of a ∼1.3 Kb band indicated with red arrows. Repeat sequences (LINE and LTR) are indicated by light gray blocks. (Results for 16day pregnant and brain were similar to 8 dL and liver respectively, data not shown).
(PDF)

Figure S6 **Real-Time-RT-PCR expression analysis of lactating mammary gland expressed genes.** Q-RT-PCR at different developmental stages in MEC isolated from mammary gland tissue of 13 week virgin (13wV), 3 days pregnant (3dP) and whole 0and 8dL MG) as well as liver from a 8d lactating animal (8dL Lvr). Real-time-RT-PCR data are normalized to K18 and expressed relative to 13wV MEC.
(PDF)

Figure S7 **ChIP-seq information.** **(A)** TableS4 H3K4me2 ChIP-seq reads used for analysis. H3K4me2 peak frequency 5 kb **(B)** and 20 kb **(C)** around TSS, in liver, Mammary epithelial cells isolated from 12 week virgin (MEC), and Lactating mammary gland tissue (Mg) based on MACs peak calling.
(PDF)

Table S1 **Primers used for ChIP q-PCR, regular ChIP-PCR, DnaseI-PCR, Bisulfite sequencing, and MassArray analysis.**
(XLSX)

Table S2 **DNA methylation levels from MassArray analysis.**
(XLSX)

Table S3 **Antibodies used for ChIP.**
(DOCX)
