# Supplementary figures and images for: Epigenetic Modifications Unlock the Milk Protein Gene Loci during Mouse Mammary Gland Development and Differentiation
Source: PLoS One. 2013 Jan 2;8(1):e53270. doi: 10.1371/journal.pone.0053270 (PMC3534698; doi:10.1371/journal.pone.0053270)

A

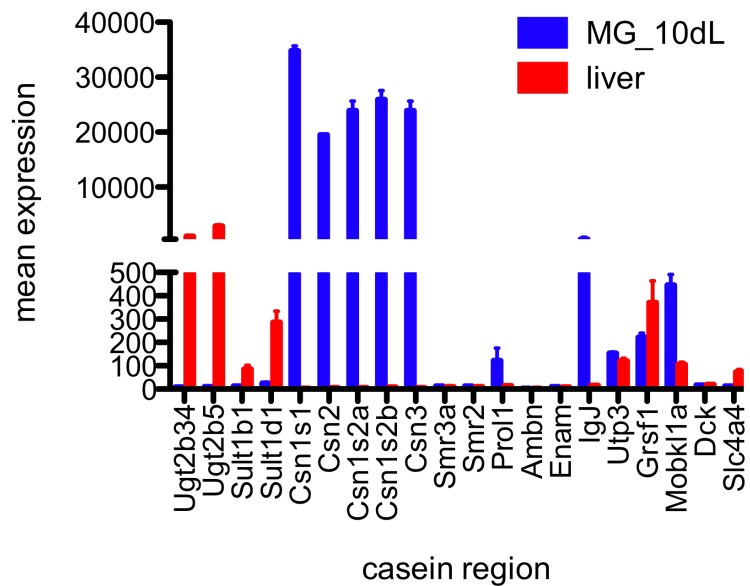

B

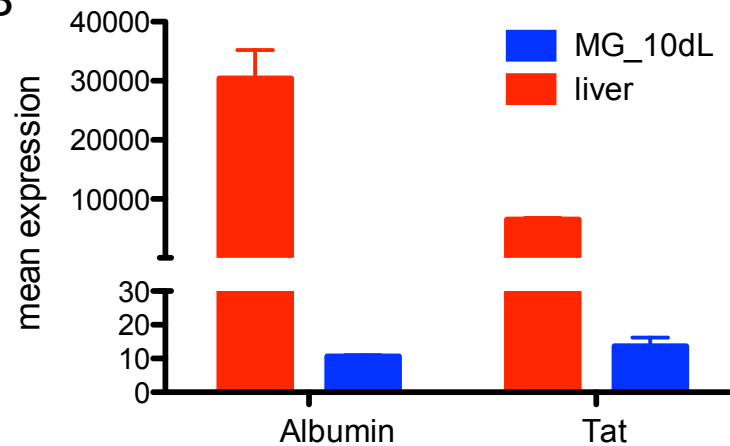

C

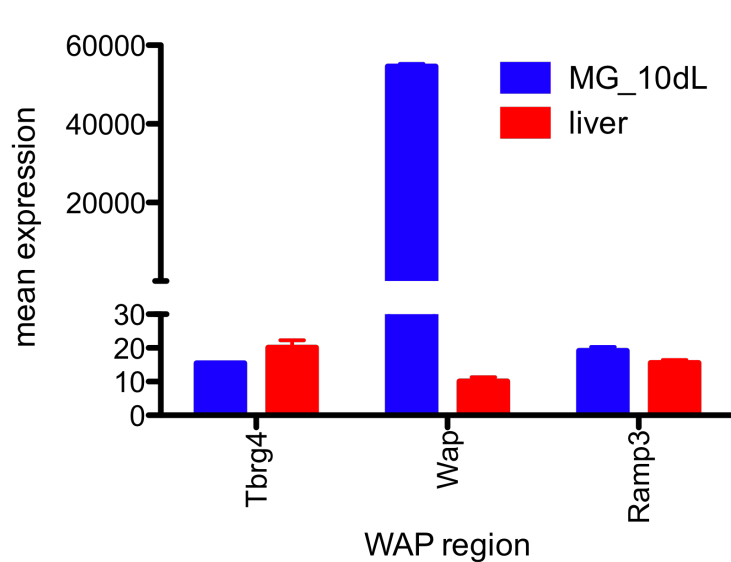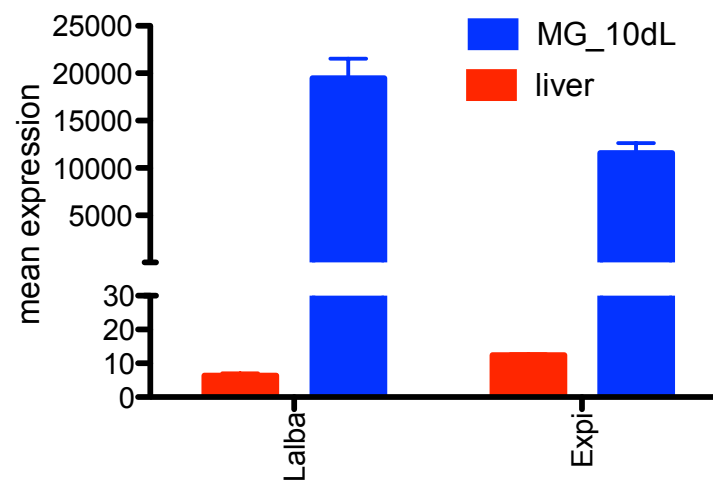

Supplement: Figure S1 — Gene expression of genes in genomic region as shown in figure 1 – 4 and S2. Expression of genes in the casein gene region (A), Albumin (ALB) and tyrosine aminotransferase (Tat) gene (B), and other milk protein gens: genes in the Whey Acidic Protein gene region, alpha-lactalbumin (Lalba), and extracellular proteinase inhibitor (Expi) (C), in liver (red) and lactating mammary gland (blue). Expression array data (GDS1805) for liver (GSM96229:, GSM96230, GSM96231) and lactating mammary gland (GSM96203, GSM96204, GSM96205) [88] were retrieved for genes in CSN and Wap regions shown in Fig. 1–4 and S2, mean values of the 3 replicates +SD were plotted in order of location of genes in genomic region as shown in figure 1–4 and S2. (PDF) [file pone.0053270.s001.pdf]

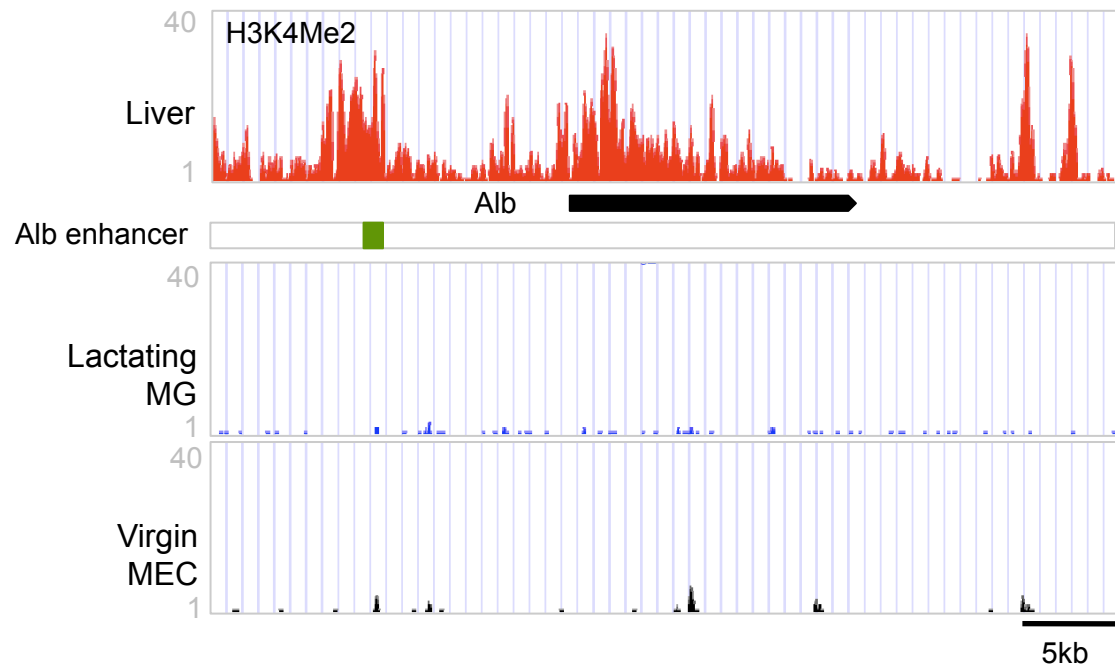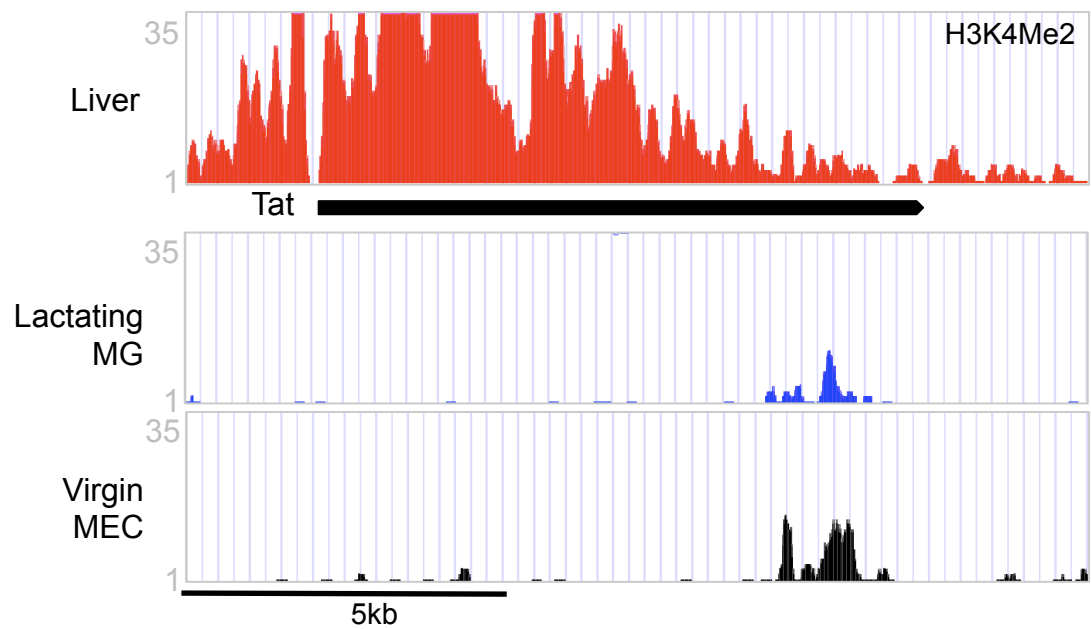

Supplement: Figure S2 — H3K4me2 ChIP-seq results for Albumin (Alb) and tyrosine aminotransferase (Tat) gene expressed in liver. ChIP-seq H3K4me2 of liver (red) lactating mammary gland (blue) and virgin MEC (black) tissue. Black arrow indicates location and transcriptional direction of Alb and Tat, Green box indicates Albumin enhancer location. (PDF) [file pone.0053270.s002.pdf]

A

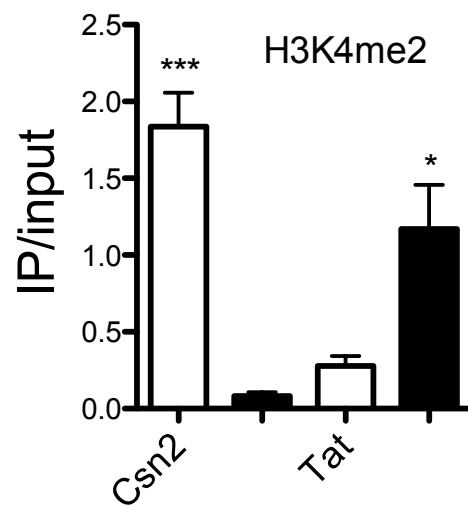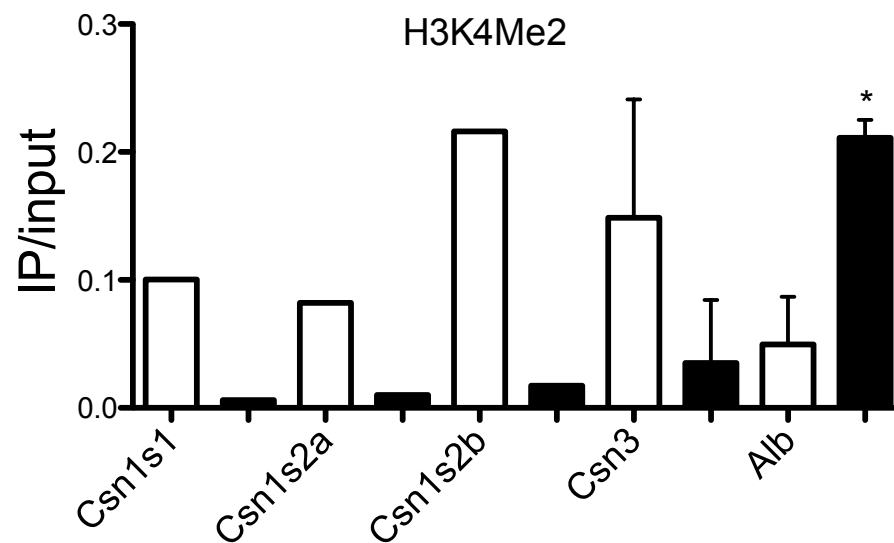

B

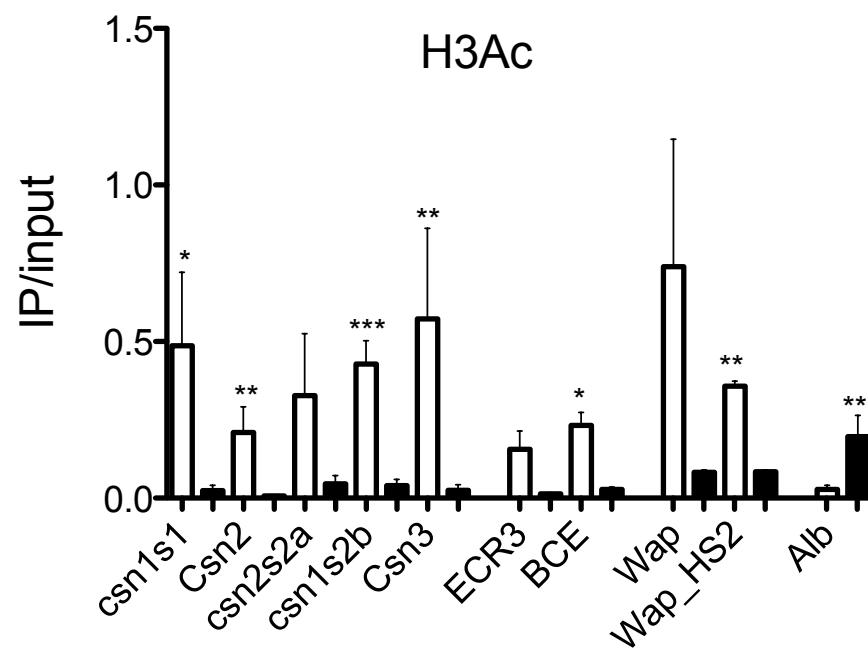

C

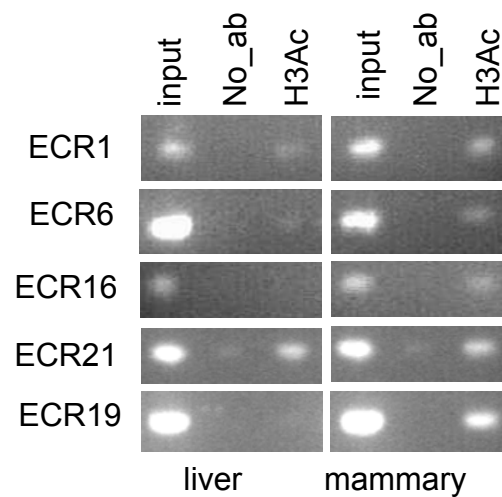

Supplement: Figure S3 — ChIP on select region in the casein gene cluster, Wap Alb and Tat genes. ChIP assays on mouse lactating mammary gland (white bars) and liver (Black bars) tissue. MG and liver chromatin was immuno-precipitated using antibodies against-acetylated-Histone-H3 and di-methylated-Lysine4 of H3 (H3K4me2), DNA was isolated, and DNA samples were analyzed using real-time PCR. (A) enrichment in samples immuno-precipitated with antibodies to (A) H3K4me2 (Csn2 n = 4; Tat n = 4; Alb n = 3; Csn3 n = 2, Csn1s1, Csn1s2a and Csn1s2b n = 1)). (B) H3Ac (Csn2, Csn3 and Alb n = 4; Csn2s1, Csn1s2a and Csn1s2b n = 3; ECR3, BCE, Wap and Wap_hss2 n = 2), compared to input (un-precipitated sample) and normalized to enrichment in the housekeeping gene GAPDH, based on real-time PCR analysis of the indicated amplicons.(P values based on 2-sample t-test are indicated; *<0.05; **<0.001; ***<0.0001) (C), representative PCR results (n = 3) of ChIP analysis of ECRs (No_Ab: no antibody control; H3Ac: anti-acetylated histone H3 antibody). Alb: albumin gene & Tat; tyrosine aminotransferase (both liver specific); Csn1s1: alpha-s1casein; Csn2: beta casein: Csn1s2a: alpha-s2a casein; Csn1s2b: alpha-s2b casein; Csn3: kappa casein; Wap-p: Whey Acid Protein promoter; Wap-H2: Wap-Hypersensitive site 2; ECR1: evolutionary conserved region 1; ECR3; ECR6; beta-casein upstream enhancer (BCE); ECR16, ECR19, ECR21 (evolutionary conserved regions). (PDF) [file pone.0053270.s003.pdf]

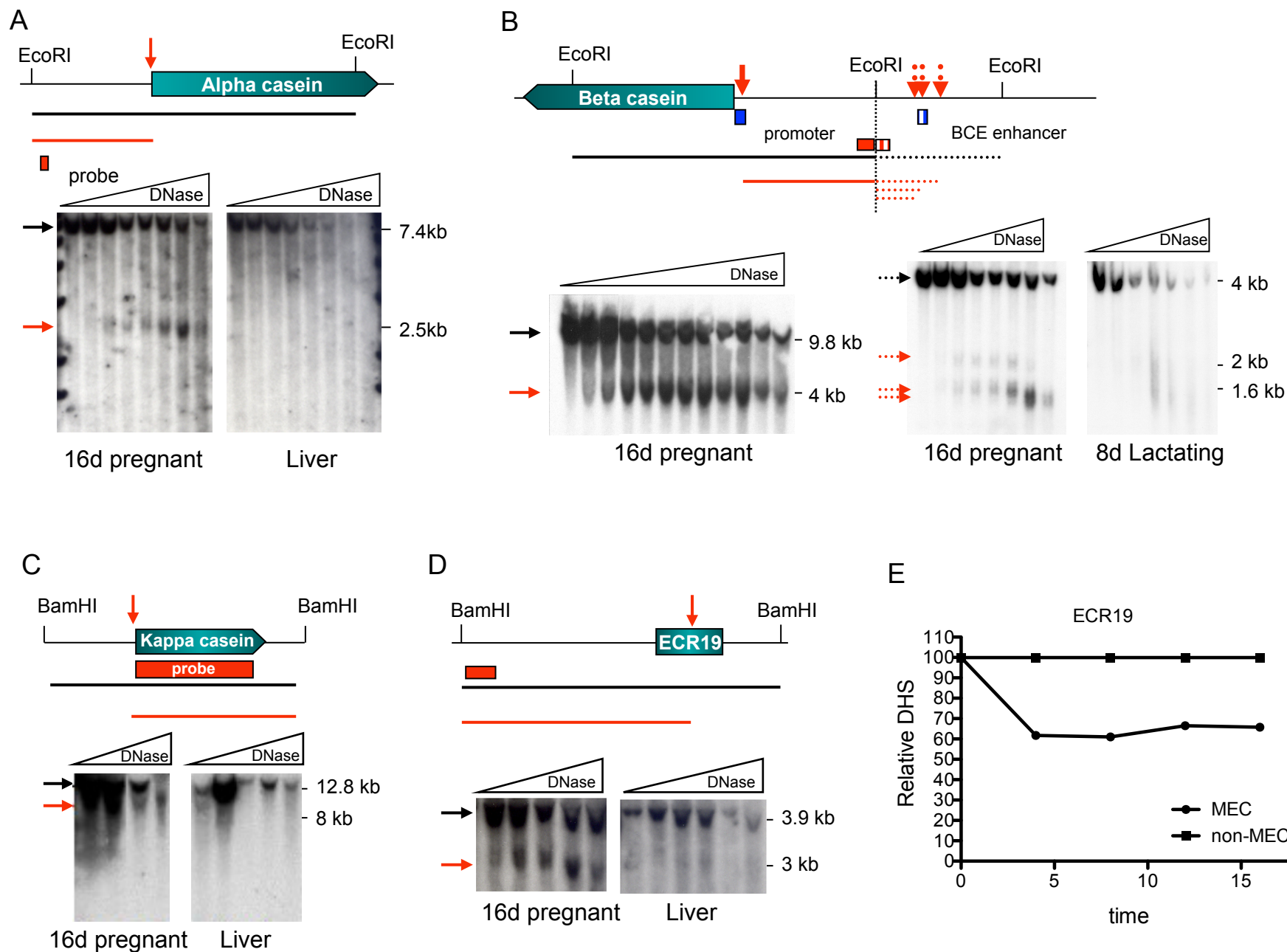

Supplement: Figure S4 — Tissue-specific DHS on casein promoters and ECRs (BCE, ECR19). (A) Csn1s1 (Alpha casein) promoter: Nuclei were isolated from 16day pregnant and liver tissue, exposed to increasing amounts of DNase1, genomic DNA isolated and digested with EcoRI. Regions in the chromatin hypersensitive to DNase1 are detected as sub-bands of the EcoRI band (red line and red arrow), size of the sub-bands indicates the location of a DNase1 hypersensitive site (vertical arrow head). Red blocks indicate location of probe used. (B) Nuclei were isolated from 16day pregnant and8 day lactating mammary gland tissue, exposed to increasing amounts of DNase1, genomic DNA isolated and digested with EcoRI. Analyzed with a probe identifying DHS at the CSn2 (beta casein) promoter (left panel) or DHS around the BCE (right panel). (C) Csn3 (kappa-casein) Nuclei were isolated from 16day pregnant and liver tissue, exposed to increasing amounts of DNase1, genomic DNA isolated and digested with EcoRI. (D) ECR19: Nuclei were isolated from 16day pregnant and liver tissue, exposed to increasing amounts of DNase1, genomic DNA isolated and digested with EcoRI. Similarly Nuclei were isolated from MEC and non-MEC cell preparations isolated from mammary glands of 8 week old virgin animals, exposed to increasing amounts of DNase1, genomic DNA isolated, PCR performed with primers flanking the HS in ECR19. Values were normalized to fragment that is not DHS in these tissues; DHS is expressed as fraction of fragments left relative to non-MEC and zero point. (PDF) [file pone.0053270.s004.pdf]

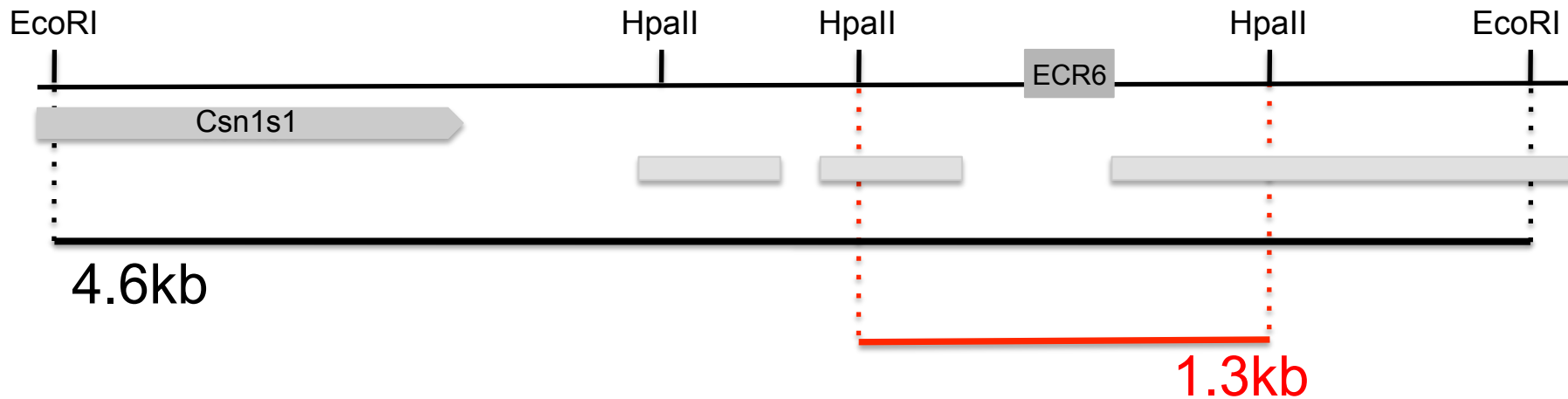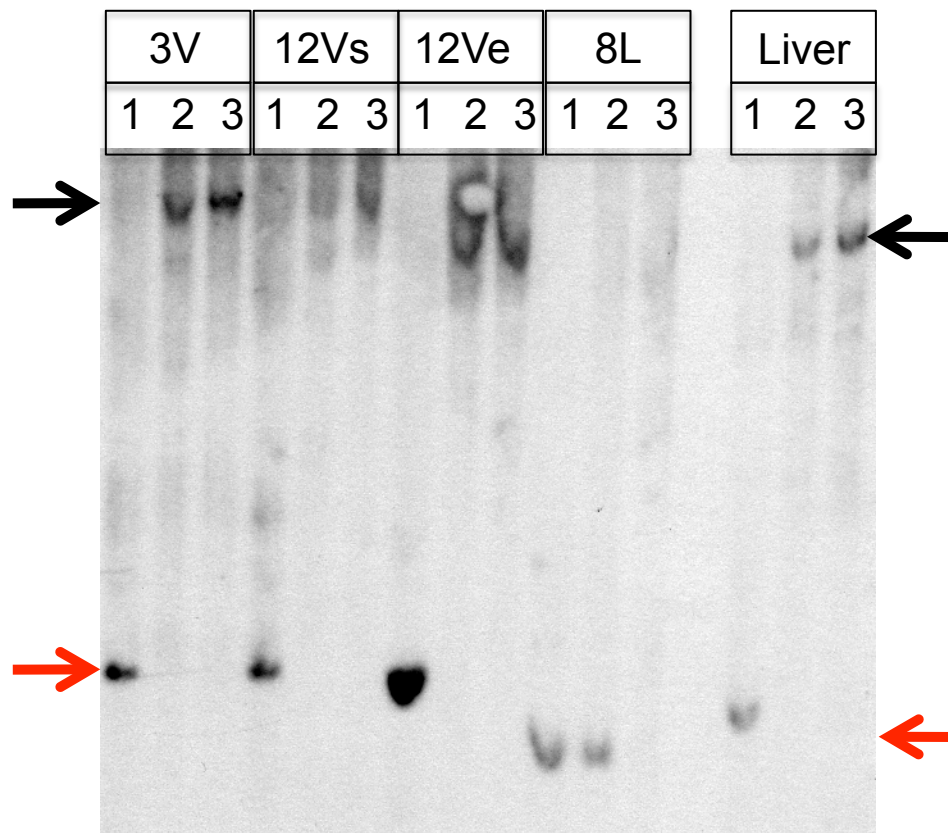

Supplement: Figure S5 — DNA methylation around ECR6 during mammary gland development and in Liver. Genomic DNA isolated from 3 week virgin (3V) mammary gland, 12 week virgin MEC (12Ve) or non-MEC (12Vs) cells, 8day lactating mammary gland and Liver was digested with EcoRI alone (lanes 3) or in combination with either MspI (lanes 1) or methylation sensitive HpaII (lane2). ECR6 sequence was used as probe in southern blot analysis. Complete digestion with MspI or HpaII results in the detection of a ∼1.3 Kb band indicated with red arrows. Repeat sequences (LINE and LTR) are indicated by light gray blocks. (Results for 16day pregnant and brain were similar to 8 dL and liver respectively, data not shown). (PDF) [file pone.0053270.s005.pdf]

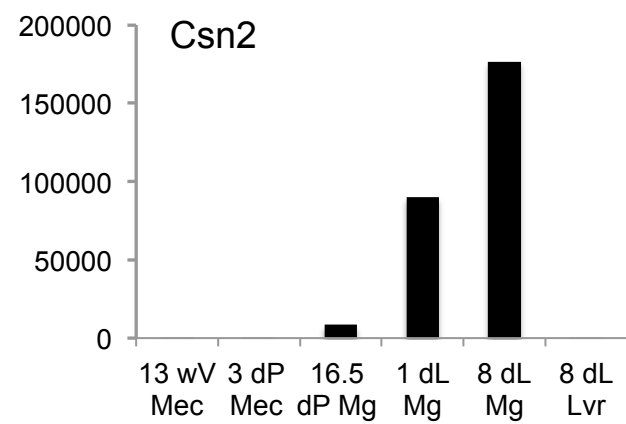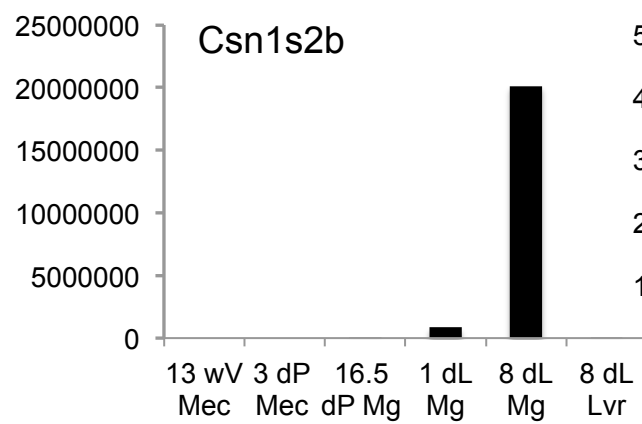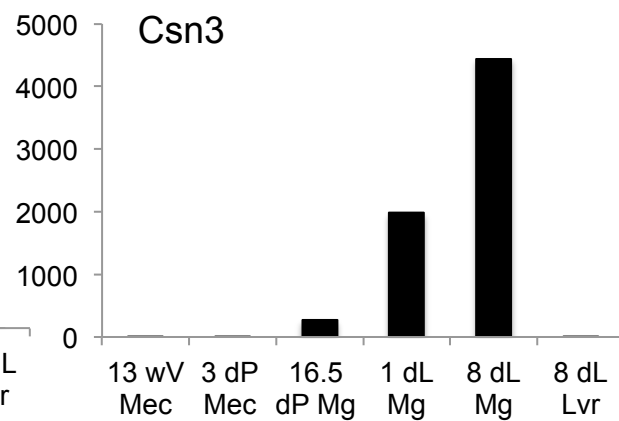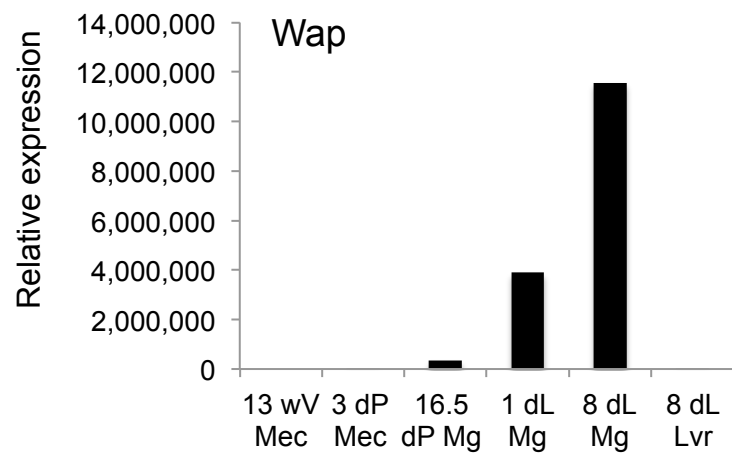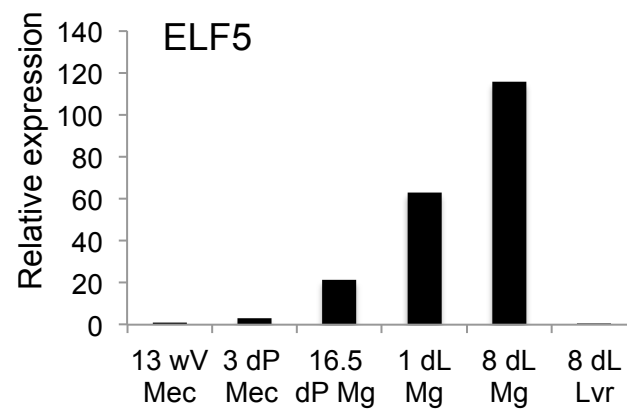

Supplement: Figure S6 — Real-Time-RT-PCR expression analysis of lactating mammary gland expressed genes. Q-RT-PCR at different developmental stages in MEC isolated from mammary gland tissue of 13 week virgin (13wV), 3 days pregnant (3dP) and whole 0and 8dL MG) as well as liver from a 8d lactating animal (8dL Lvr). Real-time-RT-PCR data are normalized to K18 and expressed relative to 13wV MEC. (PDF) [file pone.0053270.s006.pdf]
