# Supplementary material for: Epigenetic Modifications Unlock the Milk Protein Gene Loci during Mouse Mammary Gland Development and Differentiation
Source: PLoS One. 2013 Jan 2;8(1):e53270. doi: 10.1371/journal.pone.0053270 (PMC3534698; doi:10.1371/journal.pone.0053270)

(A)

Experiment	H3K4Me2<br>Uniquely Mapped (Total Reads)	Control<br>Uniquely Mapped (Total Reads)
Lvr_K4Me2	26442543	8516817
Mg_K4Me2	26137478	7930019
Mec_K4Me2	11741328	8665639

(B)

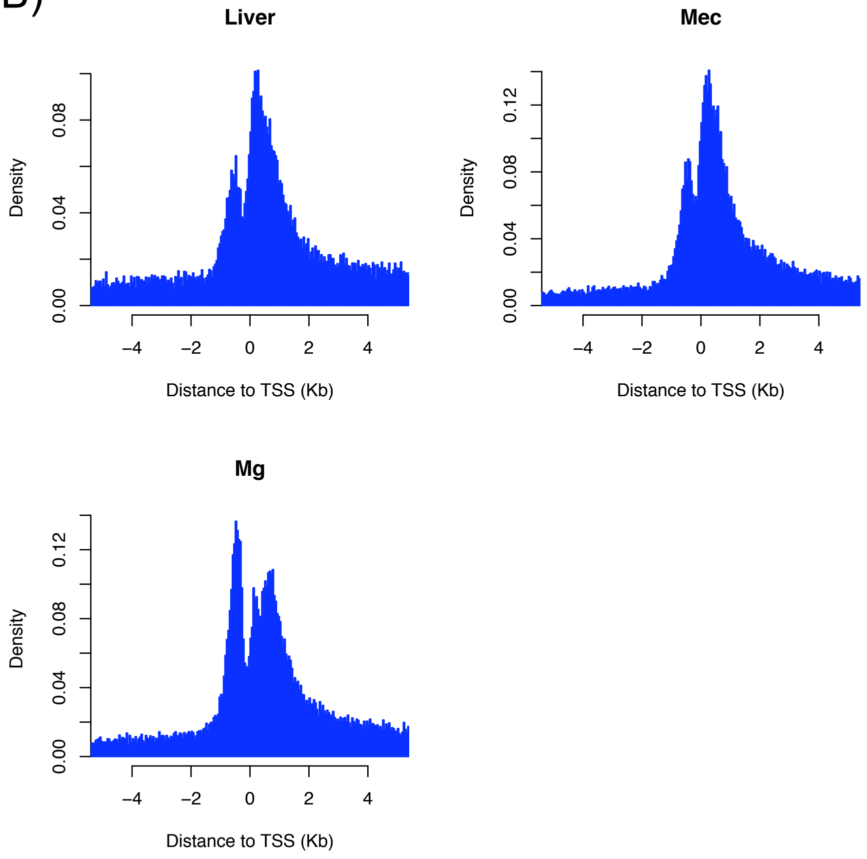

(C)

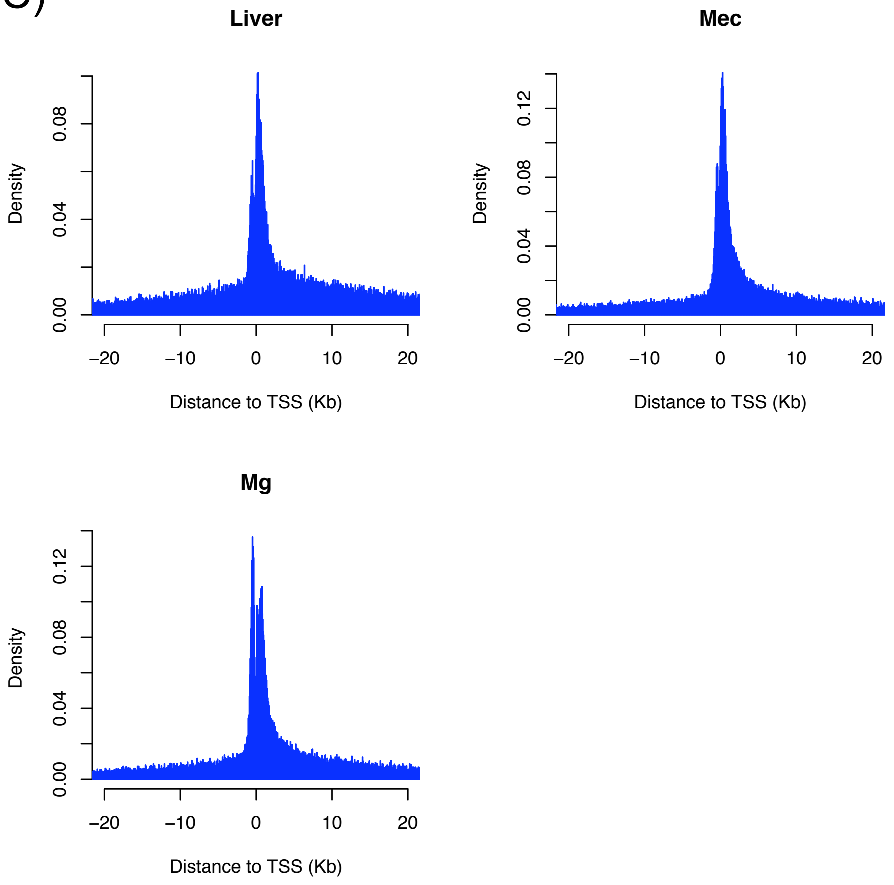

Supplement: Figure S7 — ChIP-seq information. (A) TableS4 H3K4me2 ChIP-seq reads used for analysis. H3K4me2 peak frequency 5 kb (B) and 20 kb (C) around TSS, in liver, Mammary epithelial cells isolated from 12 week virgin (MEC), and Lactating mammary gland tissue (Mg) based on MACs peak calling. (PDF) [file pone.0053270.s007.pdf]
