# Supplementary material for: Epigenetic Modifications Unlock the Milk Protein Gene Loci during Mouse Mammary Gland Development and Differentiation
Source: PLoS One. 2013 Jan 2;8(1):e53270. doi: 10.1371/journal.pone.0053270 (PMC3534698; doi:10.1371/journal.pone.0053270)
Supplement: Table S3 — Antibodies used for ChIP. (DOCX) [file pone.0053270.s010.docx]

| Histone modification | Manufacturer | Cat # | Lot# |
| --- | --- | --- | --- |
| H3K4me2 | Upstate/Millipore | 07-030 | 26335,DAM1479603, DAM1570816 |
| H3Ac (K9/14) | Upstate/Millipore | 06-599 | 29505, 25233 |
| RNAPolII | Santa Cruz | sc-899X | G1206 |

These antibodies have been used and verified in previous studies [1-3], the specificity of the H3K4me2 has been shown [4] .

references

1. Braunstein M, Sobel RE, Allis CD, Turner BM, Broach JR: **Efficient transcriptional silencing in Saccharomyces cerevisiae requires a heterochromatin histone acetylation pattern**. *Mol Cell Biol* 1996, **16**(8):4349-4356.

2. Xu CR, Cole PA, Meyers DJ, Kormish J, Dent S, Zaret KS: **Chromatin "prepattern" and histone modifiers in a fate choice for liver and pancreas**. *Science* 2011, **332**(6032):963-966.

3. Boggs BA, Connors B, Sobel RE, Chinault AC, Allis CD: **Reduced levels of histone H3 acetylation on the inactive X chromosome in human females**. *Chromosoma* 1996, **105**(5):303-309.

4. Bock I, Dhayalan A, Kudithipudi S, Brandt O, Rathert P, Jeltsch A: **Detailed specificity analysis of antibodies binding to modified histone tails with peptide arrays**. *Epigenetics* 2011, **6**(2):256-263.
